# Three novel bacteriophages isolated from the East African Rift Valley soda lakes

**DOI:** 10.1186/s12985-016-0656-6

**Published:** 2016-12-03

**Authors:** Leonardo Joaquim van Zyl, Shonisani Nemavhulani, James Cass, Donald Arthur Cowan, Marla Trindade

**Affiliations:** 1 Institute for Microbial Biotechnology and Metagenomics (IMBM), Department of Biotechnology, University of the Western Cape, Robert Sobukwe Road, Bellville, Cape Town 7535 South Africa; 2 Department of Genetics, University of Pretoria, Pretoria, 0002 South Africa

**Keywords:** Soda Lake, Bacteriophage, Myoviridae, Siphoviridae, Paracoccus

## Abstract

**Background:**

Soda lakes are unique environments in terms of their physical characteristics and the biology they harbour. Although well studied with respect to their microbial composition, their viral compositions have not, and consequently few bacteriophages that infect bacteria from haloalkaline environments have been described.

**Methods:**

Bacteria were isolated from sediment samples of lakes Magadi and Shala. Three phages were isolated on two different *Bacillus* species and one *Paracoccus* species using agar overlays. The growth characteristics of each phage in its host was investigated and the genome sequences determined and analysed by comparison with known phages.

**Results:**

Phage Shbh1 belongs to the family *Myoviridae* while Mgbh1 and Shpa belong to the *Siphoviridae* family. Tetranucleotide usage frequencies and G + C content suggests that Shbh1 and Mgbh1 do not regularly infect, and have therefore not evolved with, the hosts they were isolated on here. Shbh1 was shown capable of infecting two different *Bacillus* species from the two different lakes demonstrating its potential broad-host range. Comparative analysis of their genome sequence with known phages revealed that, although novel, Shbh1 does share substantial amino acid similarity with previously described *Bacillus* infecting phages (Grass, phiNIT1 and phiAGATE) and belongs to the Bastille group, while Mgbh1 and Shpa are highly novel.

**Conclusion:**

The addition of these phages to current databases should help with metagenome/metavirome annotation efforts. We describe a highly novel *Paracoccus* infecting virus (Shpa) which together with NgoΦ6 and vB_PmaS_IMEP1 is one of only three phages known to infect *Paracoccus* species but does not show similarity to these phages.

**Electronic supplementary material:**

The online version of this article (doi:10.1186/s12985-016-0656-6) contains supplementary material, which is available to authorized users.

## Background

Soda lakes are sodium carbonate (Na_2_CO_3_)-dominated environments with varying salinity and high pH values, usually between 9 and 11, but occasionally greater than pH 12 [1–3]. These lakes are found in arid and semi-arid areas where high evaporation rates facilitate the accumulation of salts in local depressions and due to the high buffering capacity of sodium carbonate, soda lakes are the only habitats that maintain stable high alkalinity [4–6]. The best studied lakes are those of the East African Rift Valley (EARV) which have been scientifically documented for many decades, two of which, Lake Shala (LS) and Lake Magadi (LM) are located in Ethiopia and Kenya respectively [7–11]. The EARV lakes are situated in an environment of active volcanism and differ from other soda lakes as surrounding hot springs supply water to the lake depressions, whereas others are supplied by the leaching of rainfall through the surface into the lake basins [3].

Soda lakes are the most biologically productive non-marine aquatic environments known [10, 12] and although the microbial composition of these lakes has been well studied [11, 13–16], little is known about their viral populations [17, 18]. It is now accepted that bacteriophages (phages) are the most abundant biological entities in most ecosystems and soda lakes are no exception, with studies conducted on Mono Lake placing viral abundance at 10^9^ ml^-1^, among the highest in natural aquatic environments [19]. Although much is known about phages that infect certain groups of microorganisms (*Mycobacterium*, *Staphylococcus*, *Escherichia*, *Pseudomonas* and *Lactococcus*), much work is needed to expand on our knowledge of phages infecting other hosts [20, 21]. The impact that phages have on higher trophic levels is also becoming clearer as elegantly demonstrated using a soda lake environment as model [22, 23]. Although this study was a demonstration of the direct effect phage infection can have on a bacterial population and higher trophic structures, they are also thought to shape populations through genetic exchange [24, 25] and by so doing affect the biogeochemical cycles present. The study of phages from these environments has also highlighted some unique viruses such as ΦCh1, an archaeal virus which carries both DNA and RNA [26]. Therefore, there is a need to better understand the viral composition in these, and other environments.

Here, we describe phages that infect *Bacillus*- and *Paracoccus* species. Several studies have highlighted the importance of these microbes in biogeochemical cycling in soda lakes [27]. The Firmicutes make up a substantial portion (11%) of the microbial community in Lonar Lake and *Bacillus* species, together with *Methylomicrobium* and *Methylophaga* species, and were shown to be the dominant methylotrophs in this Lake [28]. They’ve also been shown to be responsible for metal speciation and mobilization of arsenic in Mono Lake [29] while some members of the Firmicutes such as *Bacillus alkalidiazotrophicus*, play a role in the nitrogen cycle [30]. *Rhodobacteraceae* in turn were shown to be one of the dominant families in many soda lakes (Bogoria, Lonar, Zabuye and Kauhako), and the most diverse Family in Ethiopian soda lakes [16]. In particular, *Paracoccus* species (family *Rhodobacteraceae*) have been identified as part of the denitrifying community [29]. Although several prophages have been identified in *Paracoccus* species’ genomes, only two phages (NgoΦ6 and vB_PmaS_IMEP1), are known to infect *Paracoccus* species [31]. Thus, to better understand the diversity and biology of bacteriophages and their potential effects on their hosts, in particular from haloalkaline environments, we isolated and characterized three phages from EARV soda lakes including a novel phage infecting a *Paracoccus* species*.*

## Methods

### Sampling, Bacterial isolation and culturing

Medium A (broth and agar) was used for bacterial isolation and culturing [32]. Medium A broth contained 1% of glucose, 0.5% of peptone, 0.5% of yeast extract, 5% of NaCl, 0.1% of K_2_HPO_4_ and 0.02% of MgSO_4_.7H_2_O. Medium A agar was prepared from medium A broth with the addition of 1.5% of bacteriological agar. Soft agar used in plaque assay required 0.75% of bacteriological agar. All components were dissolved in water and then adjusted to pH9 using 10 N NaOH. Unless stated otherwise, all strains were cultured at 37 °C. Bacteria were isolated for this study from soil sediments of both LM and LS stored at 4 °C. Ten grams of sediment from each sample were suspended in 100 ml of medium A broth and diluted 10 fold with water. One hundred microlitre volumes of each dilution were spread on medium A agar plates, which were incubated for 24 h at 37 °C. After visual inspection of bacterial isolates for varying colony morphologies, colonies were picked from each plate. Bacterial strains were stored in medium A broth containing 50% glycerol at -80 °C until required. Isolates ERV9 and HS3, which are part of the IMBM strain collection, were previously isolated from LS sediment on medium A adjusted to pH9. One gram of soil from each site was serially diluted in 1 × PBS (phosphate buffered saline) and dilutions (10^-2^–10^-7^) were plated. Plates were incubated for 8–10 weeks at 37 °C.

### Phage isolation and assays

Fifty grams of each soil sediment was mixed with 100 ml of medium A broth and incubated at 37 °C on a shaking platform at 120 rpm for 24 h. Fifty millilitre aliquots were removed and centrifuged at 5000 × *g* for 15 min. The suspensions were filtered first through a 0.45 μm, followed by 0.22 μm syringe filter. The filtrates were used for phage-host infection test plaque assays [33] using two-layer agar plates. The soft agar layer contained 100 μl of mid-log cultures of the newly isolated bacteria mixed with 100 μl of filtrate. Plates were incubated at 37 °C for 24 h. A single plaque was picked using a sterile 1 ml pipette tip and sub-cultured using the same host strain. This phage purification process was repeated 3 times. After phage purification, phage stocks were stored in medium A containing 50% glycerol at -80 °C for long term storage.

One step growth curves were determined as described by [34] with slight modification. Bacterial host strains were cultured overnight in 5 ml of medium A broth at 37 °C at 120 rpm on a shaking platform. Two hundred microliters of each overnight culture was inoculated in 50 ml of medium A broth and incubated at 37 °C at 120 rpm on a shaking platform until the cell density of the cultures reached approximately 1x10^8^ CFU/ml. One millilitre aliquots of each bacterial culture were mixed in microfuge tubes with 0.1 multiplicity of infection (MOI) (MOI = Plaque forming units (pfu) of virus used for infection/number of cells) of phage, in triplicate, and incubated at 37 °C at 120 rpm on a shaking platform for 10 min allowing the phage to adsorb to the bacterial host. Cells were centrifuged at 6000 × *g* for 10 min to remove the unadsorbed phage. Supernatants were removed and the pellets were resuspended in 1 ml of medium A broth. Fifty microliters of the resuspended cultures were transferred to 50 ml of medium A and mixed well. A one millilitre aliquot of each culture was transferred into a microfuge tube (time was noted as T = 0) and the rest (±49 ml) of the triplicate cultures were incubated at 37 °C with aeration (120 rpm) on a shaking platform. Samples were taken every 30 min for 6.5 h. Plaque forming units (PFU) were determined by the plaque assay.

Phages were prepared for TEM visualization as previously described [35]. Phage lysates (10 ml) were centrifuged at 25,000 × *g* for 1 h in an Eppendorf 5417R centrifuge. Supernatants were discarded and the pellets were suspended in 1 ml of 0.1 M ammonium acetate solution, then incubated at 37 °C at 120 rpm on a shaking platform for 16 h to allow the phage pellets to resuspend. Centrifugation and resuspension steps were repeated twice. Each phage suspension was resuspended in a final volume of 20 μl 0.1 M ammonium acetate after the last washing step. TEM images were taken with an FEI Tecnai F20 Field Emission Gun operated at 200 kV at the University of Cape Town’s Electron Microscopy Unit. Two microliters of each phage suspension were placed onto a carbon coated copper grid (200 mesh), washed with distilled water and stained with 2% uranyl acetate. The samples were observed at 50 000 X magnification.

### DNA extraction, PCR and sequence analysis

Approximately 100 ml of phage lysate was filter sterilized using 0.45 μm followed by 0.22 μm syringe filters. This was followed by addition of 7.5 ml 20% (wt/vol) PEG8000 to every 30 ml of phage lysate and overnight storage at 4 °C. Phage lysates were centrifuged at 13,000 × *g* for 30 min. The supernatants were discarded and the pellets resuspended in 1 ml SM buffer. SM buffer was prepared using 20 ml of 5 M NaCl, 8.5 ml of 1 M MgSO_4_, 50 ml of Tris-HCl (pH 7.5) and 10 ml of 1% gelatin solution. A 5 μl volume of DNAseI at 1 mg/ml and 5 μl of RNAseA at 12.5 mg/ml concentration were added to 1 ml volume of phage suspensions in SM buffer to remove bacterial DNA and RNA. The reactions were incubated at 37 °C for 30 min. Following the addition of 10 μl of Proteinase K at 10 mg/ml and 20 μl of 20% SDS, the reactions were incubated at 55 °C for 1 h to allow for the disruption of the phage capsids. Phenol/chloroform DNA extraction was used to extract phage DNA. An equal volume of phenol:chloroform:isoamyl alcohol (25:24:1) was added to the supernatants, and the reactions were centrifuged at 13,000 × *g* for 5 min. The upper layer was transferred to new tubes, and the process of adding phenol:chloroform:isoamyl alcohol, centrifugation and removal of the upper layer was repeated. An equal volume of chloroform:isoamyl alcohol (24:1) was mixed with the upper layer containing the sample and centrifuged. The upper layer was transferred into 1.5 ml eppendorf tubes. A 45 μl volume of 3 M sodium acetate (pH5.2) and a 500 μl of 100% isopropanol were added, and the solution was incubated overnight at -20 °C to precipitate the DNA. The pellet was collected by centrifugation at 14,000 rpm for 20 min. The DNA pellet was washed twice using 1 ml of 70% ethanol. The DNA pellet was air dried at room temperature, resuspended in 30 μl of 1 X TE buffer and stored at -20 °C.

The 16S rRNA gene amplification was carried out using universal bacterial primers E9F 5’-GAGTTTGATCCTGGCTCAG-3’ [36] and U1510R 5’-GGTTACCTTGTTACGACTT-3’ [37] to identify bacterial isolates. The PCR mix included 5 μl of 10X DreamTaq buffer, 5 μl of 2 mM dNTPs, 2 μl of both 1 mM forward and reverse primers, 100 ng of DNA and 1.25 U DreamTaq Polymerase. Each reaction was adjusted to a final volume of 50 μl with nuclease free water and amplified in an automated thermal cycler (Thermo Hybaid). The PCR conditions were: initial denaturation at 95 °C for 3 min, followed by 35 cycles of denaturation at 95 °C for 30s, annealing at 55 °C for 30s and extension at 72 °C for 1 min, with a final extension at 72 °C for 10 min. DNA fragments of approximately 1500 bp were generated and visualised by electrophoresis on a 1% agarose gel.

Sanger sequencing was performed using an ABI PRISM® 377 automated DNA sequencer at the Central Analytical Facility of the University of Stellenbosch (South Africa). For 16S rRNA sequencing, primers E9F and U1510R were used. Next generation sequencing was performed using an Illumina MiSeq sequencer using the Nextera XT library preparation kit (Illumina) and a 10% phiX v3 spike as per the manufacturer’s instructions (Preparation Guide, Part #15031942 Rev A May 2012) as well as the MiSeq Reagent kit V2 (500 cycle). One nanogram of uncloned, unamplified viral DNA was used to prepare one Nextera XT library with each phage barcoded for de-multiplexing after sequencing. Sequencing was performed at the Institute for Microbial Biotechnology and Metagenomics (IMBM), University of the Western Cape, Cape Town, South Africa. The raw reads were trimmed (bases with a Q-score less than 36 were trimmed from the 3’end) and de-multiplexed at the sequencing facility generating 2 × 250 bp reads, resulting in a set of paired (read pairs, forward and reverse) fastq files per phage.

Sequences were analysed using BioEdit Version 7.0 software and DNAMAN Version 4.13. The NCBI database was used for analysis of DNA sequences and homology searches. The Basic Local Alignment Search Tools (BLAST) programme was used to determine sequence similarity and identity to known sequences in the GenBank database using software from the National Centre for Biotechnology Information (www.ncbi.nlm.nih.gov/). *De novo* assembly of phage genomes was performed using CLC genomics workbench ver. 6.5 (CLC bio, Denmark). Annotation of phage genomes was performed by manual BLASTp searches of ORFs predicted by CLC as well as BLASTx searches for regions in which no ORFs were predicted. The complete genome sequence of all three phages is available on the GenBank database under accession numbers KR072689 (Shpa), KU640380 (Shbh1) and KU665491 (Mgbh1).

The software program TETRA was used to perform tetranucleotide usage deviation analysis [38, 39]. tRNA genes were predicted using the tRNAscan-SE program (http://tinyurl.com/snbk2). Direct repeats were identified using REPFIND (http://tinyurl.com/zkh2pnc) with a 15 bp minimum repeat length. Inverted repeats were identified using UGENE (http://ugene.unipro.ru) using 20 bp minimum and 80% similarity as search parameters. Codon usage data for *P. denitrificans* was obtained from (http://tinyurl.com/zeqorau). Transmembrane regions were predicted using the TMHMM server (http://www.cbs.dtu.dk/services/TMHMM). Protein repeats were identified using the RADAR server (http://tinyurl.com/pcpves3). CRISPR sequences were identified through BLAST searches using the full length genome sequence against the CRSIPR database (http://crispr.u-psud.fr). The phylogenetic tree for terminase sequences from various phages and bacteria was created using MEGA6 [40]. All positions with less than 95% site coverage were eliminated. That is, fewer than 5% alignment gaps, missing data, and ambiguous bases were allowed at any position. There was a total of 239 positions in the final dataset.

## Results and Discussion

### Isolation of phages and basic characterization

Seven bacterial isolates (Table 1) from the IMBM culture collection, previously isolated from LS and LM were used to screen for phages. On challenging the isolates with sediment from both lakes clear plaques were produced on isolate HS3 with LS sediment and isolate MGK1 showed clear plaque formation with sediment from both lakes. Following single plaque purification, three distinct phages were identified and named Mgbh1 (MGK1—LM sediment; clear plaques), Shbh1 (MGK1—LS sediment; clear plaques) and Shpa (HS3—LS sediment; clear plaques). The phages were tested for their ability to infect the seven bacterial isolates from both lakes. Mgbh1 and Shbh1 infected MGK1. Shbh1 can additionally infect isolate ERV9 forming turbid plaques with characteristic “bulls eye” morphology, whereas Shpa could only infect HS3. This suggests that Shbh1 could be a broad-host range phage which promotes genetic exchange between hosts. “Bulls eye” plaques on ERV9 also suggests lysogeny of the host whereas it may be lytic on MGK1.

**Table 1:** Bacterial strains employed for phage isolation

Strain (Lake Source)	pH, NaCl % (wt/vol)	Closest 16S rRNA BLAST hit (sequence length bp/%)	Phage That Infects
Shala1 (Shala)	9, 8	*Halomonas axialensis* (1395/98)	*-*
Shala2 (Shala)	9, 7	*Bacillus licheniformis* (1422/99)	*-*
Shala3 (Shala)	9, 7	*Virgibacillus salarius* (1426/99)	*-*
Shala4 (Shala)	9, 8	*Halomonas venusta* (1378/99)	*-*
MGK1 (Magadi)	9, 6	*Bacillus halodurans* C-125 (1398/97)	Mgbh1, Shbh1
ERV9 (Shala)	9, 6	*Bacillus pseudofirmus* OF4 (1414/99)	Shbh1
HS3 (Shala)	10, 6	*Paracoccus marinus* (1399/97)	Shpa

Phage preparations from all four isolations were visualized by TEM. Morphologically Shbh1 belongs to the family *Myoviridae* while Mgbh1 and Shpa belong to the family *Siphoviridae* (Table 2).

**Table 2:** Summary of phage characterization data including bright field TEM images of phages. The scale bar is 100 nm in length

Phage source	Host	Bacteriophage	Genome size (bp)	Phage family	TEM image	No. of ORFs	G + C content (%)	Dimensions (nm)		Burst size per cell	Eclipse (h)	Latent period (h)	Rise time (h)
								Approximate head diameter	Approximate tail length				
LM	MGK1	Mgbh1	58951	*Siphoviridae*		80	45.4	49	200	882	1	4	3
LS	MGK1	Shbh1	138081	*Myoviridae*		178	36.4	92	226	1550	0.5	3.5	3
	ERV9	Shbh1	138081	*Myoviridae*		178	36.4	92	226	106	1	3.5	2.5
LS	HS3	Shpa	38261	*Siphoviridae*		56	64.7	37	200	1722	0.5	4	3.5

**Fig. 1:**
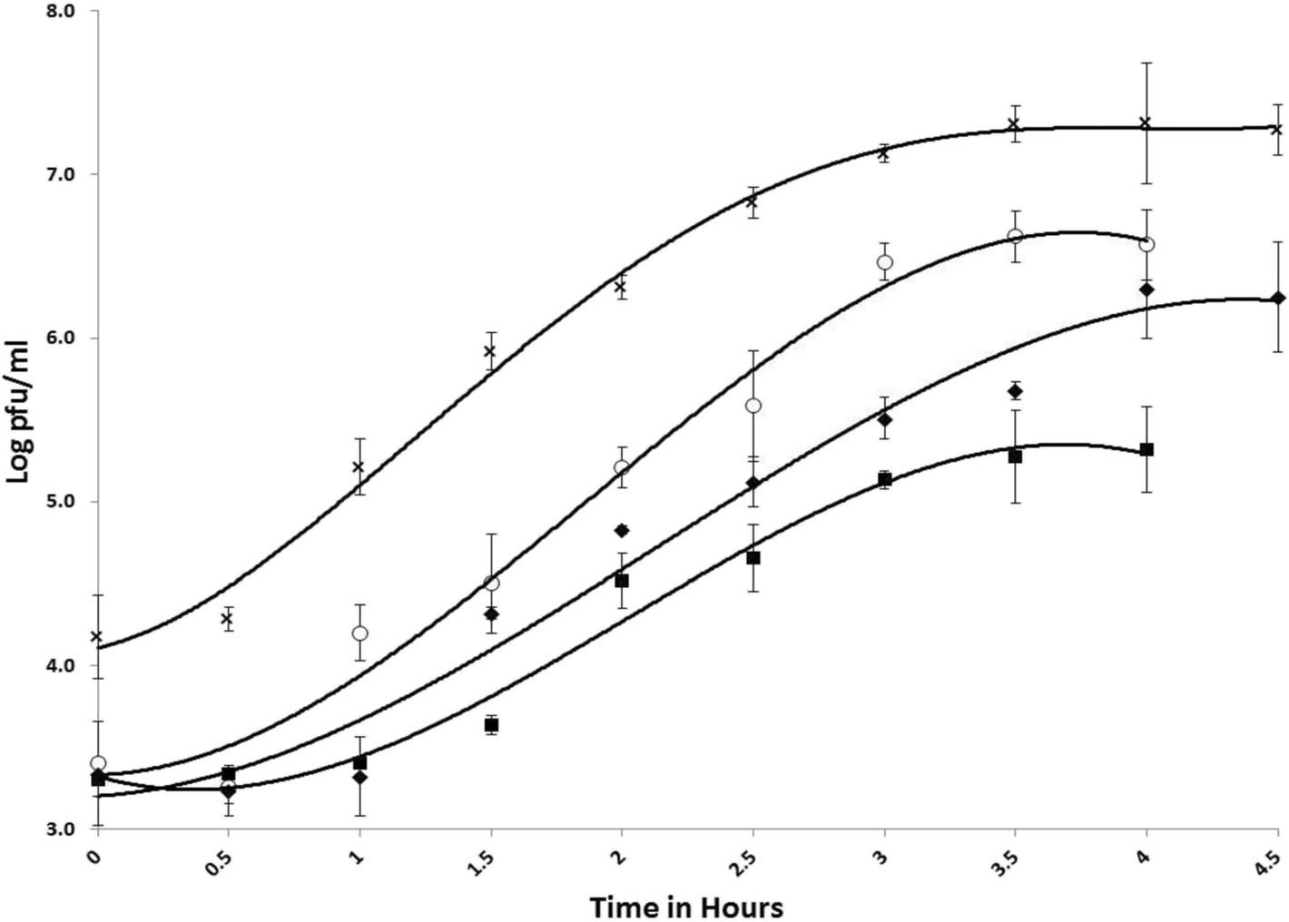
One step growth curves of phages Shpa (✖) Mgbh1 (♦) Shbh1 on MGK1 (◯) and Shbh1 on ERV9 (■)

One step growth curve data showed that Mgbh1, Shpa and Shbh1 (with MGK1 as host), had large burst sizes producing >800 particles per cell (Fig. 1 and Table 2). Shbh1 had a much smaller burst size when using ERV9 as host, which it likely lysogenizes and could explain the reduced burst size. It is noteworthy that none of the phages displayed a particularly acute burst, but rather a burst drawn out over 2.5 to 3 h and suggests that phage release from single infected cells could be asynchronous [41, 42]. Turbid or clear plaques for Shbh1 on the two different hosts, could be the result of different transcription/translation rates as cultures grew at more or less the same rate according to their growth curves (not shown). This could be influenced by the growth conditions used (37 °C and a fixed salt concentration and medium composition). As only one set of conditions was employed, the phage may behave differently in the two hosts.

### Phage genomes

#### Genome composition and phylogeny

**Fig. 2:**
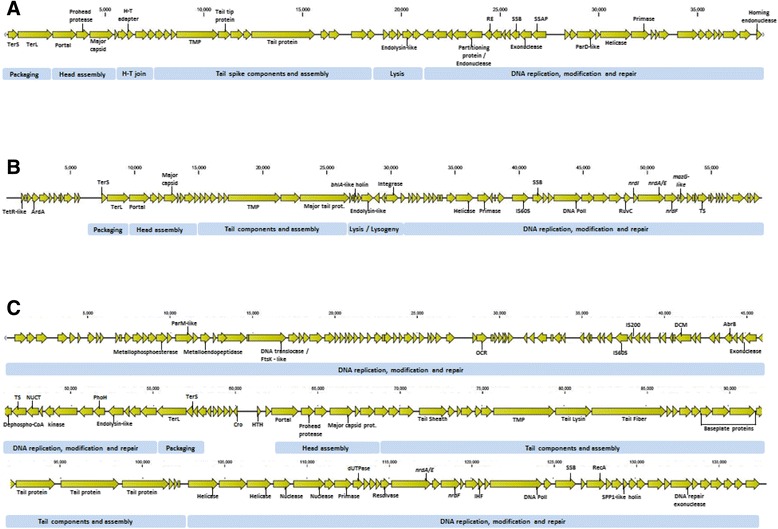
Genomic arrangement of Shpa (**a**), Mgbh1 (**b**) and Shbh1 (**c**). Blue boxes indicate modular areas

The phage genomes varied in size ranging from 58.9 kb to 138 kb (Table 2). The phage genomes all display the modular arrangement well documented in other phages from these families (Fig. 2). Shpa shares little overall nucleotide similarity with any phage on the NCBInr database with the highest identity being to small portions (38–394 bp at 75 to 92%) of *Paracoccus, Rhodobacter* and *Rhizobium* species’ genomes. More distantly related is a section (±2.8 kb at 67% identity; 2576–5387 bp), encoding the major capsid protein and a protease, to the *Silicibacter* species TM1040 genome (CP000377.1). This region of the *Silicibacter* species genome encodes a putative prophage. The phage genomes appear to be compact, with only 9%, 12% and 13% non-coding regions in Shpa, Mgbh1, and Shbh1 respectively.

Mgbh1 shares a region of ±3 kb (49269–52282 bp at 67% identity) with the *Bacillus subtilis* subspecies *spizizenii* W23 genome (CP002183.1) and much shorter sections (29–64 bp at 100%) with the *B. halodurans* C-125 genome sequence (BA000004.3). The ±3 kb area encodes the ribonucleotide reductase subunits and the nucleotide similarity is likely due to the flanking genomic region coding for a prophage as well as the need to conserve RNR function and regulation [43]. Shbh1 does share significant nucleotide similarity with well-known *Bacillus* species *Myovirus* phages namely SIOphi (70% identity over 32% of the genome), phiNIT1, phage Grass and to a lesser extent the more recently described phage, phiAGATE [44, 45]. Neither Mgbh1 nor Shbh1 appears closely related to the only other sequenced alkaliphic *Bacillus* infecting phage BCJA1c [46] with only one open reading frame (ORF31 on Mgbh1) showing highest sequence identity to a homologue on that phage genome. The genome sequence of two *Idiomarinaceae* infecting phages Phi1M2-2 (NC_025471; 36844 bp) and 1 N2-2 (NC_025439; 34773 bp), isolated from LM, has been determined. However, none of the phages described here show similarity at the nucleotide or amino acid level to these viruses. Nucleotide sequence alignment of Shbh1 with several of its closest relatives show some conservation at the nucleotide level (Additional file 1: Figure S1). Notably, the first ±45000 bp shows little similarity to any of its relatives, with the exception of a block from ±10000 bp to ±17000 bp which contains a SpoEIII homolog. It also shares some similarity to the ORFs encoding structural proteins in related phages. However, there are four regions that show little or no homology to the structural proteins of closely related phages: 75678–76596 bp, 82861–86954 bp, 91464–95056 bp, 98815–102490 bp. The first of these regions lies in the tail tape measure protein, and likely reflects the differences in tail length between Shbh1 and its relatives. The other three regions occur in a region coding for putative tail fiber proteins and two other tail proteins without defined function. These difference may reflect that a unique cellular feature is targeted by the phage for binding to the host or may reflect the adaptation to a haloalkaline environment.

The G + C content of Shpa is in the same range as for its host (*Paracoccus* species G + C range 63.4–70.4%), while the G + C content for Shbh1 is slightly lower than for *B. halodurans* (43.7%) or *B. pseudofirmus* (40.3%) which is often observed for phage-host pairs [47]. In the case of Mgbh1 the G + C content is slightly above that of its host, *B. halodurans*. On average it is found that phages have a G + C content 4% lower than their hosts [47], with fewer examples of phage with G + C content higher than their host. Deviation in G + C content between phages and their hosts has been used to indicate that the phage does not regularly infect, or has evolved in a particular host, as G + C content tends to ameliorate over time between phages and their hosts [48, 49]. This, together with the potential broad-host range nature of the phage suggests that Shbh1 may not prefer infecting *in situ* the hosts on which it was isolated here. While other factors such as the availability of a suitable *attB* site and regulation of gene expression will play a part in whether or not the phage is lytic or lysogenic on a particular host, it is worth noting that the G + C difference for Shbh1 compared with *B. halodurans* is greater than between Shbh1 and *B. pseudofirmus*. This agrees with the observation of Rocha that lytic phages are often higher in AT content than lysogenic phage compared with the host. Tetranucleotide usage deviation (TUD) analysis gave a value of 0.64 when comparing Shbh1 to both host genomes (*B. halodurans* and *B. pseudofirmus*). The correlation coefficient was 0.65 when comparing Shbh1 and Mgbh1 to each other. The TUD value was 0.46 when comparing the Mgbh1 genome to that of MGK1. This lower TUD correlation perhaps together with a slightly higher G + C content, for Mgbh1, could suggest that the host used here may not be the host that this phage has regularly infected or has co-evolved with for a long time. TUD analysis gave a Pearson’s correlation coefficient of 0.74 and 0.69 when comparing Shpa to the two sequenced *Paracoccus* genomes.

**Fig. 3:**
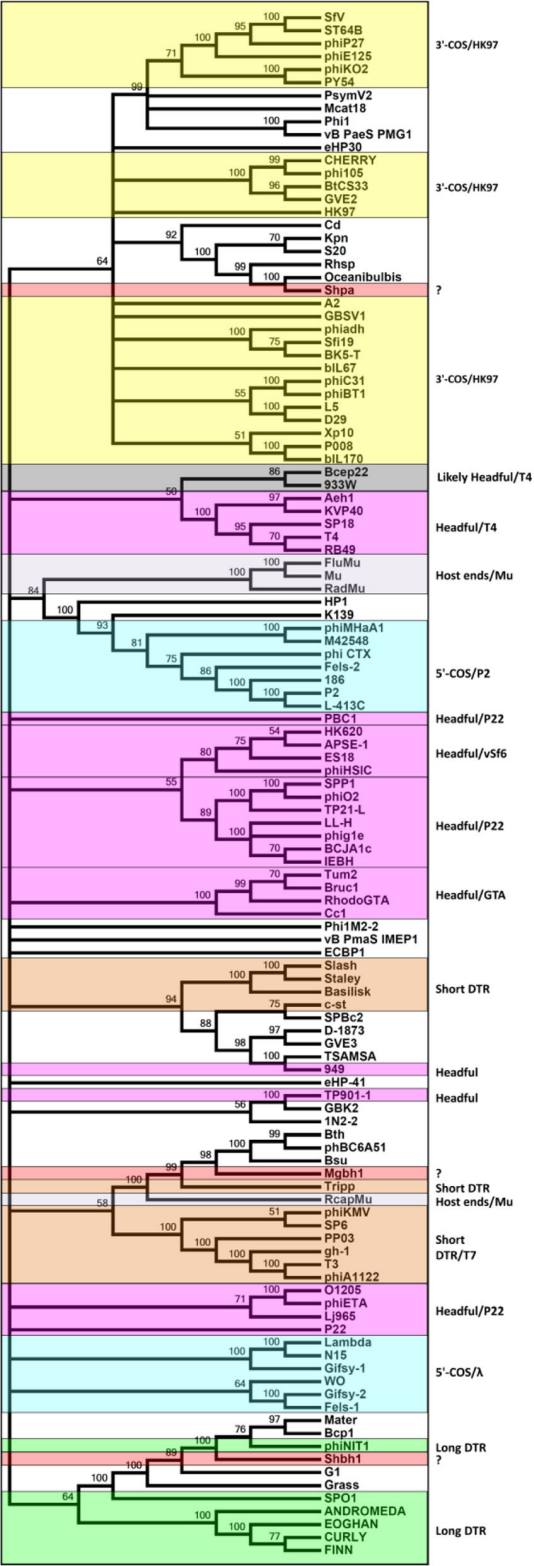
A condensed (50% cutoff) neighbor-joining tree of 117 large terminase subunit amino acid sequences from tailed phages and bacteria. The percentage of replicate trees in which the associated taxa clustered together in the bootstrap test (1000 replicates) is shown next to the branches [70, 71]. The names of the phages or bacterial sequences are shown at the right of each branch. Coloured boxes demarcate related groups of terminases with similar packaging strategies. Accession numbers for aligned sequences are: Bcp1 - YP_009031337; Bth - WP_063675351; phBC6A51 - NP_852525; Grass - YP_008771417; phiNIT1 - YP_008318309; Oceanibulbis- KZY05261; vB_PmaS_IMEP1 - YP_009126425; eHP-41 - AFH23043; ECBP1 - YP_006908844; RcapMu - YP_004934675; Kpn - WP_048270899; Rhsp - WP_011840500; Tripp - YP_009210523; Bsu - CUB50907; G1 - YP_240892; Mater - YP_009151157; Mcat18 - AKI27764; SfV - NP_599034; Phi1 - ALY08144; Cd - YP_001686870; Phi1M2-2 - AIM40760; 1 N2-2 - AIM40704; eHP30 - AFH22611; S20 - YP_007673310; ES18 - YP_224140; phiHSIC - YP_224236; HK620 - NP_112076; APSE-1 - NP_050979; LL-H - YP_001285878; phig1e - NP_695170; Lj965 - NP_958579; O1205 - NP_695104; phiETA - NP_510934; P22 - AAA72959; T3 - NP_523347; phiA1122 - NP_848309; gh-1 - NP_813786; PP03 - WP_010953264; phiKMV - NP_877482; SP6 - NP_853601; Tum2 - WP_035256432; Cc1 - NP_421586; RadMu - NP_285417; FluMu - P44224; Bruc1 - AAL52531; RhodoGTA - AAF13179; phiKO2 - YP_006582; PY54 - NP_892047; ST64B - NP_700375; phiP27 - NP_543088; phiE125 - NP_536358; HK97 - NP_037698; A2 - NP_680484; Sfi19 - NP_049926; BK5-T - NP_116494; phiadh - CAB52518; P008 - AAY97804; biL170 - AAC27181; biL67 - NP_042313; Xp10 - NP_858953; phiC31 - NP_047924; phiBT1 - NP_813693; L5 - NP_039677; D29 - NP_046828; Bcep22 - NP_944278; T4 - NP_049776; RB49 - NP_891724; Aeh1 - NP_944105; KVP40 - NP_899601; phiCTX - NP_490600; P2 - NP_046758; L-413c - NP_839851; 186 - NP_052251; Fels-2 - YP_001718747; HP1 - NP_043485; K139 - NP_536648; 933 W - ; Mu - NP_050632; SP18 - YP_003934803; TP901-1 - NP_112694; Lambda - NP_040581; N15 - NP_046897; WO - BAA89640; Gifsy-1 - YP_001700612; Gifsy-2 - YP_001700675; Fels-1 - YP_001700563; vB_PaeS_PMG1 - YP_005098205; PsymV2 - YP_009017589; Shpa - AKG94514; Shbh1 - AMQ66579; Mgbh1 - AMQ66673; Basilisk - AGR46580; SPO1 - YP_002300330; PBC1 - YP_006383455; EEOGHAN - YP_007517399; FINN - YP_007517630; ANDROMEDA - YP_007517474; CURLY - YP_007517553; BCJA1c - YP_164412; IEBH - YP_002154374; CHERRY - ABA46388; phi105 - ADF59135; BtCS33 - YP_006488672; GVE2 - YP_001285808; Slash - YP_008771935; Staley - YP_008770747; D-1873 - EES90358; vB_BanS_Tsamsa - YP_008873483; 949 - YP_004306307; SpBc2 - NP_046608; c-st - YP_398598; phiMHa1 - YP_655470; M42548 - AGK02301; SPP1 - NP_690654; TP21-L - YP_002333563; GVE3 - YP_009223720; GBSV1 - YP_764473; phiO2 - YP_008240332; GBK2 - YP_009010473

The terminase large subunit identified on Mgbh1 shows highest similarity to proteins from *Bacillus* (phBC6A51) and *Paenibacillus* (Tripp) phages (Fig. 3 and Additional file 2: Table S1) when performing a BLASTp search against only *Caudovirales* sequences on the NCBI database. However, it is most closely related to many terminase sequences found in genome sequences of a variety of *Bacillus* species when searching against the NCBInr database and Metavir classification identifies *Bacillus subtilis* phage Grass as the closest relative. This would suggest that Mgbh1 is related to *Bacillus* prophages rather than lytic phages which infect these hosts. It may also point to the large amount of information missing from current databases, and the biological relationship described should help to more accurately describe the phylogeny of many unknown phages.

A small ORF just upstream of the putative Mgbh1 TerL shows weak similarity to two terminase small subunits (TerS) on the NCBI database using a BLASTp search against all classified and unclassified *Caudovirales* sequences from *Lactobacillus* phages phiJL-1 and phi jlb1. It also has a helix-turn-helix motif, involved in DNA binding, similar to other terminase small subunits [50]. The protein also models well, when using the automated model building feature in SWISSMODEL, with an rmsd value of 0.157 Å for Cα to 2AO9. 2AO9 is a homo 18-mer of a protein from *B. cereus* phage phBC6A51, which also lies directly upstream of its TerL. Taken together, this suggests that ORF13 can tentatively be assigned the small subunit terminase for Mgbh1. The putative large terminase subunit (ORF98) from Shbh1 is most closely related to those from *Bacillus* myoviruses Grass, phiNIT1, Bcp1, vB_BceM-Bc431v3 and clusters with phages from the *B. cereus* group (Fig. 3 and Additional file 3: Table S2). Metavir identified phiAGATE as the closest relative, with PHAST suggesting phage Grass as the closest relative. A small ORF (136aa) is present directly upstream of ORF98 but could not be identified as the terminase small subunit based on BLASTp searches alone, showing how different these proteins are from their characterized homologues. The read coverage of Shbh1 indicates that the genome has long terminal repeats indicated by a region of higher than (3234x) average coverage (1714x) from ±26700 bp-30500 bp, while PAUSE3 analysis indicated two points of significant read build up in this region at bases 29638 bp and 30054 bp.

When compared with the NCBInr database, Shpa’s putative terminase large subunit (Fig. 3 and Affitional file 4: Table S3) is most closely related to a terminase sequence of a prophage on an *Oceanibulbus* species (77% identity over 99% of the sequence) genome which, like *Paracoccus*, belongs to the family *Rhodobacteraceae*. For Shpa, BLASTp searches against the NCBInr database also returned hits to terminase-like sequences in non-*Paracoccus* bacterial genome sequences, rather than terminase-like sequences in prophages of *Paracoccus* species. Twenty-six *Paracoccus* genomes have been sequenced thus far including a *Paracoccus halophilus* but only one (vB_PmaS_IMEP1) of their phages has been sequenced. This suggests that the phage described here does not infect a wide range of *Paracoccus* species or has not encountered the hosts sequenced thus far and, to date, appears to be unique to this environment. The closest match when comparing it to just viral sequences is to *Tetraselmis viridis* (phycodnavirus) infecting virus S20 (Fig. 3) and it also shows little similarity with the terminase from the only other described *Paracoccus* phage, vB_PmaS_IMEP1. S20 is thought to be a phage of bacteria which co-culture with algae [51].

Based on the analysis presented in Fig. 3 we predict that Shpa could have 3’ overhanging cohesive ends, while Shbh1 terminase groups with myoviruses for which a mechanism has yet to be defined (SPO1, phage GRASS and phiNIT1) and Mgbh1 likely uses a headful mechanism.

#### Structural proteins

Major capsid and portal proteins could be identified for all three phages, while head to tail joining proteins could be identified in Shpa and Mgbh1. Phage Shbh1 encodes putative tail fiber proteins (ORF126, 135 and 137) which contain repeated protein sequences similar to what has been identified in the long and short tail fiber proteins (gp34 and gp12) of phage T4 [52]. As Shbh1 is not too dissimilar from mesophilic *Bacillus* infecting phages, it’s structural proteins may give insight into the adaptation of proteins in general or specifically phage structural proteins to high pH, high salt environments. Similarly, ORFs 28, 29 and 30 of Mgbh1 show repeated protein sequences and likewise a 129 bp direct repeat was identified close to the C-terminal of ORF19 in Shpa that is responsible for a repeated amino acid sequence. ORF19 encodes a putative tail fiber protein which ends with the stop codon at 15621 bp. However, homology to a choice-of-anchor A domain protein (gp21 *Burkholderia* phage phi644-2, YP_001111100.1, 1101 bp) starts immediately downstream (15622 bp) of the stop codon in reading frame 3 and continues until 16330 bp. It may be that read through translation results in the full length protein being produced.

#### Mobile genetic elements

An intact IS605 element (ORF53) was identified on Mgbh1, but did not have an IS200 element associated with it. A 33 bp direct repeat was identified 90 bp upstream (39260–39293; 39326–39359) of the IS605 element on Mgbh1. IS605 and IS200 elements identified on Shbh1 appear truncated and likely inactive. No repeats of significance could be identified in the immediate vicinity of the insertion sequence elements on Shbh1. The location of the IS elements suggests that these are areas which tolerate disruption to a certain degree (DNA replication and repair) possibly due to these functions being complemented by host factors and similarly suggests that areas related to expression of proteins involved with phage structural components are more sensitive to interruption.

#### Nucleotide metabolism, replication and gene expression

Ribonucleotide reductases (RNR) were identified in both Mgbh1 and Shbh1. An activator protein could also be identified on Mgbh1 (ORF 62; *nrdI*), but not on Shbh1. This suggests that the RNR encoded on Mgbh1 belongs to class Ib while the one on Shbh1 belongs to class Ia RNRs. The separation of the two subunits of RNR by a homing endonuclease such as is identified on Shbh1 has been described before, although in this case does not appear to interrupt the reading frame of either subunit [53]. No RNRs were detected on Shpa.

Mgbh1 and Shbh1 both encode thymidylate synthases. It has been demonstrated that TS1 type thymidylate synthases are unique to the Bastille group of *Bacillus* infecting phages and may be used as a phylogenetic marker [54]. According to the identity cut-offs defined by Asare, the thymidylate synthase of Shbh1 (57% identity to that of Bastille and E-value of 1 × 10^-111^) demonstrates that it belongs to the Bastille group, whereas Mgbh1 does not. Shbh1 however appears to lack a dihydrofolate reductase homolog, often found between two and six ORFs downstream of the thymidylate synthase, in Bastille phages, which was also identified as another marker gene for this group. It also does not encode a metal dependent beta-lactamase homolog as has been found for the other members of this group, but does encode two putative metal dependent enzymes: ORF13 and 20, encoding a metallophosphoesterase and metalloendopeptidase membrane proteins respectively. The Shbh1 SpoIIIE homolog (ORF 25), another hallmark of this group of phages, is located eight and five ORFs downstream of these metallo-enzymes respectively which is an arrangement also observed in other Bastille phages.

Both Mgbh1 and Shbh1 encode DNA polymerases (ORF 57 and 160), whereas Shpa does not. Both of these enzymes should have 3’-5’ exonuclease activity, however the DNA polymerase identified on Shbh1 also contains an N-terminal uracil DNA glycosylase (UDG) domain similar to that found on the *Bacillus* phage SPO1 DNA polymerase. Although characterized as a part of DNA excision repair processes, the presence of this domain in DNA polymerases has been suggested to aid in polymerase processivity [55]. ORFs likely involved with DNA replication of Shpa are ORFs 33, 34, 38, 45 and 46, which include a helicase, primase, single stranded DNA binding protein and a protein containing a ParBc endonuclease-like domain [56].

GC skew analysis of Shbh1 shows a possible replication origin (global minimum in GC skew) and terminus (global maximum in GC skew) at ±60kbp and 0/138 kbp respectively (Additional file 5: Figure S2). An intricate inverted and direct repeat structure at the ±60kbp site also suggests a replication or transcription regulation site at this position. The genome of Shbh1 is terminally redundant with 336 bp direct terminal repeats found at the ends of the sequence. A second local maximum (±30kbp) corresponds to an area where the direction of transcription changes. A putative replication origin and terminus was also identified for Shpa at the 0/38kbp and at ±21kbp respectively (Additional file 6: Figure S4). The GC skew plot for Mgbh1 is similar to those of genetic elements which replicate solely through a rolling circle mechanism, while those that go through both theta and RC stages show defined minima [57] (Additional file 7: Figure S3). No repeats of significance could be identified for Mgbh1.

ORF17 on Mgbh1 does not have an identifiable start codon, however the remainder of the protein appears to be related to phage proteins on the NCBI database, which are also described as partial proteins. It may be that ORF16 and ORF17 are co-translated. ORF27 and 45 on Mgbh1 may have earlier start sites (16911 bp, 33220 bp) and be produced through stutter or read through translation. The Imm_39 domain containing protein on Shbh1 has a stop codon in the reading frame and the full length protein may be produced as a result of read through translation. Three 27 bp direct repeats were identified on Shpa from 17204 bp to 17284 bp which may serve as a transcription regulation site.

No tRNAs could be identified for Shbh1 or Mgbh1. It has been suggested that a reason for the presence of, especially large numbers of tRNAs, on broad host range phage genomes is to compensate for different codon usage patterns in host bacteria. Shbh1 infects at least two different *Bacillus* species, yet harbours no tRNAs, suggesting very similar codon usage profiles in these hosts (9.8% mean difference in codon usage). Shpa encodes one tRNA (Trp; 37819–37889 bp). An analysis of the proteins encoded by Shpa shows that the tryptophan content is 1.3 times as high as for proteins in *P. denitrificans*, giving a possible reason as to why this tRNA is retained on the phage [58].

#### Lysis and Lysogeny

ORF107 on Shbh1 appears to be a *cro*–like regulator and identifies the area upstream of it, which includes inverted and direct repeats, as a region likely involved in control of the lysis/lysogeny balance. The “bulls eye” plaque morphology for Shbh1 on ERV9 is suggestive of the phage being lysogenic in this host, however there was no easily identifiable integrase on Shbh1. No integrase related proteins could be identified on Shpa either. There is however a resolvase (ORF152) and a recombinase (ORF164), on Shbh1, which may serve as integrase for the phage. A holin-like protein (ORF167) was identified on Shbh1, but separated from the endolysin-like protein (ORF94) by 77 kbp and appears to lack the dual start motif described for other holins [59]. This holin-like protein has two predicted transmembrane regions (24–46aa; 56–75aa), classifying this as a ClassII holin [60].

Two holin-like proteins (ORF33 and 34) identified on Mgbh1 lie directly upstream of a putative endolysin (ORF35). ORF34 has a potential dual start motif (MESEIIRM), however the methionine residues are separated by more residues than usual. Both of the Mgbh1 proteins only have one predicted transmembrane region (ORF33—5-24aa; ORF34—12-34aa) each, grouping them with ClassIII holins.

It is of interest that the terminase large subunit of Mgbh1 is most similar to sequences which are likely from lysogenic phages in the genome sequences of *Bacillus* species, but not to terminases of *Bacillus* infecting phage genome sequences deposited on the NCBI database. Together with the observation that clear plaques are formed on the MGK1 host, this could suggest that the phage is a lytic version of a phage(s) that, more often than not, display a lysogenic lifestyle.

A 65 bp nucleotide sequence (29421–29486 bp) with 100% DNA identity to a sequence adjacent to the *B. halodurans* 5S rDNA was identified on Mgbh1 located inside ORF37 just upstream of the putative recombinase/integrase (ORF38). This may suggest that the phage inserts itself at this position on the *B. halodurans* chromosome when lysogenizing the host.

A holin could not be identified on Shpa, however a peptidoglycan recognition protein (PGRP) domain protein (ORF27) was identified, and it is known that endolysin-like proteins have such domains [61, 62]. It also displays homology to lysozyme-like proteins likely involved in cell wall degradation, either during infection or cell lysis for release of progeny. Shpa seems to lack the genes associated with lysogeny (integrase, recombinases), and together with the observation of clear plaques on HS3 suggests that it too is lytic.

#### Phage signatures on currently available genomes and metagenomes

A BLASTn search of Mgbh1 against the CRISPR database identified three perfectly conserved spacer regions on the genome sequence of *B. halodurans* C-125, and are all part of the same CRISPR array (Table 3). This suggests that Mgbh1 or a very closely related phage could have infected *B. halodurans* strains isolated from various regions around the world (C-125 from soil in Japan) [63]. Six other imperfect match spacers were also identified in the C-125 genome. The nucleotide sequence differences between these spacers and phage genome sequence could represent adaptation of the phage to host resistance. It has been shown that even single nucleotide changes in proto-spacer regions incorporated by bacteria as CRISPR spacers can result in phage becoming virulent again [64]. For Shbh1 two potential CRISPR spacers could be identified on the *B. halodurans* C-125 genome (Table 3). No CRISPR spacers could be identified for Shpa, when searching current databases, and this is likely due to there being few *Paracoccus* genomes on these databases (including the host species identified here) or could indicate that the phage is not widespread.

**Table 3:** CRISPR spacer regions corresponding to the genome sequence of Shbh1 and Mgbh1 in currently available bacterial genomes

Phage	Bacterial strain	CRISPR spacer sequence	CRISPR repeat unit sequence	Location on phage
Mgbh1	*B. halodurans* C-125	CAAAGTGATCGGATCATTCGTTTAATCCCTCCCCTT	GTCGCACTCTTCATGGGTGCGTGGATTGAAAT	47980–48015
		TCGAAAGTTTCGAGGAGTCTAACGGAGCGGAAGA	GTCGCACTCTTCATGGGTGCGTGGATTGAAAT	16247–16280
		CTAACTGCAACGTTATATACGCGGGCTGTCTTGA	GTCGCACTCTTCATGGGTGCGTGGATTGAAAT	21267–21296
		GTTGC^G^_T_ ACCGCTCCTTGCGGCGACGTGTCGGGC	GTCGCACTCTTCATGGGTGCGTGGATTGAAAT	5105–5138
		ATTATCGTTAGGGTAGGGGTTACTA^T^_C_^G^_T_ AGATGAC	GTCGCACTCTTCATGGGTGCGTGGATTGAAAT	24974–25007
		GTGCGGTAGATGATCC^A^_G_ GACTT^T^_C_ GATCGCCGTAATT	GTCGCACTCTTCATGGGTGCGTGGATTGAAAT	35921–35956
		CGCCCA^C^_T_ TTACGAATAACAGCAAG^A^_G_ ACGTGCATAA	GTCGCACTCTTCATGGGTGCGTGGATTGAAAT	48859–48893
		CCTACCGC^G^_C_ CGCCA^C^_T_ GTCGTTAA^A^_G_ TCCTCTAGCGTC	GTCGCACTCTTCATGGGTGCGTGGATTGAAAT	27504–27539
		GGATTTTACGA^G^_A_ AC^A^_G_ TGGAGCGATATATAA^A^_C_ CGCG	GTCGCACTCTACATGAGTGCGTGGATTGAAAT	3543–3577
Shbh1	*B. halodurans* C-125	TCTTAA^A^_G_ TCTTTTAT^C^_T_ AATTGCTCTCTTGTAAC^A^_G_	GTCGCACTCTACACGAGTGCGTGGATTGAAAT	34484–34516
		^G^_A_ AAGTATGA^C^_T_ AAGTTTGTAGG^G^_T_^C^_A_ TTGAAAATTCATG	GTCGCACTCTTCATGGGTGCGTGGATTGAAAT	25969–26003

Subscript sequence corresponds to phage DNA sequence while superscript corresponds to the sequence of the bacterium

Two soda lake metagenome datasets (Kulunda Steppe Russia and Soda Lake California) are currently available and these were investigated for the presence of the phages described here [65] (SRR3306837). From the Soda lake metagenomic dataset 111 reads mapped to the Shpa genome at 80% similarity and 60% length fraction or reads, covering 2771 bp of the genome, and 34 reads mapped to Shbh1. This could indicate the presence of these phages or related phages in this environment. There were no sequences similar to either Mgbh1 or Shbh1 identified in the Kulunda Steppe metagenome, however two contigs showed regions with nucleotide similarity to the terminase large subunit from Shpa (1613 bp; 68% identity with 97% query coverage). When a BLASTx was performed of these sequences against the NCBInr database, the best hit was within a *Rhodobacter sphaeroides* genome. To further investigate whether or not the contigs represented phages or prophages, these were analysed by PHAST. Neither contig appears to contain more than just the one ORF with similarity to the large terminase subunit and neither appears to be a phage or prophage, making it unlikely that these represent related phages. Thus, this suggests that Shpa may not be present in that environment.

## Conclusion

Here we’ve described three novel phages isolated from halo-alkaline environments, and as very few phages that infect alkaliphic and more specifically haloalkaliphilic microorganisms have been described, these three phages are rather unique [46, 66, 67]. It is unknown exactly what the role of the host organisms identified here is in their respective environments, however the presence of possibly lytic phages such as Shpa and Shbh1, could have a significant impact on their hosts and therefore the environment in which they find themselves. The impact that phages can have in natural settings is well understood, but their impact in anthropogenic settings is less clear, where they could either be the cause of a problem or be a solution for it. *Paracoccus* species have been isolated from a wide range of environments, including biofilters used in the treatment of waste gases from animal rendering plants, groundwater contaminated with dichloromethane, a sulfide-oxidizing, denitrifying fluidized-bed reactor, wastewater from semiconductor manufacturing processes and in and around child care facilities [68, 69]. One aspect to the study of phages and their interactions with their hosts is the potential for phage derived products and novel genetic systems. These can be in the form of phage host pairs (phage display, genetic systems, phage therapy; food preservation), endolysin/lysozyme (phage therapy; biofouling), DNA/RNA polymerases, DNA/RNA ligases for research and includes the chance of discovering new interactions such as CRISPR systems which have been developed into excellent genome editing tools [70]. The endolysin proteins of all three phages could be useful in treating fouling of filters by these or closely related organisms and may perform best in high pH/high salt environments. Although soda lakes make up a small percentage of aquatic environments, the microorganisms which are found in these environments can help us further delineate the limits at which life occurs and the addition of these three phages to the databases will hopefully aide in this process.

## Additional files

Additional file 1: Figure S1. Whole genome alignment of phage Shbh1 with six of its closest relatives. Similarly coloured regions indicate homology or local collinear blocks (LCB) between nucleotide sequences, with the level of similarity indicated by the height of the bars within each LCB. Genome alignments were performed using MAUVE. (DOCX 136 kb)
Additional file 2: Table S1. Predicted open reading frames on Shpa and closest BLASTp hit on the NCBI database. (DOCX 20 kb)
Additional file 3: Table S2. Predicted open reading frames on Mgbh1 and closest BLASTp hit on the NCBI database. (DOCX 23 kb)
Additional file 4: Table S3. Predicted open reading frames on Shbh1 and closest BLASTp hit on the NCBI database. (DOCX 37 kb)
Additional file 5: Figure S2. GC skew analysis of the Shbh1 genome showing putative replication origin (*ori*) and termination sites (*ter*) calculated using a window size of 1000 bp and a step size of 100 bp. (DOCX 23 kb)
Additional file 6: Figure S4. GC skew analysis of the Shpa genome showing putative replication origin (*ori*) and termination sites (*ter*) calculated using a window size of 1000 bp and a step size of 100 bp. (DOCX 22 kb)
Additional file 7: Figure S3. GC skew analysis of the Mgbh1 genome showing putative replication origin (*ori*) and termination sites (*ter*) calculated using a window size of 1000 bp and a step size of 100 bp. (DOCX 22 kb)
