# Supplementary material for: Three novel bacteriophages isolated from the East African Rift Valley soda lakes
Source: Virol J. 2016 Dec 3;13:204. doi: 10.1186/s12985-016-0656-6 (PMC5135824; doi:10.1186/s12985-016-0656-6)
Supplement: Additional file 1: Figure S1. — Whole genome alignment of phage Shbh1 with six of its closest relatives. Similarly coloured regions indicate homology or local collinear blocks (LCB) between nucleotide sequences, with the level of similarity indicated by the height of the bars within each LCB. Genome alignments were performed using MAUVE. (DOCX 136 kb) [file 12985_2016_656_MOESM1_ESM.docx]

 **Figure S1.** Whole genome alignment of phage Shbh1 with six of its closest relatives. Similarly coloured regions indicate homology or local collinear blocks (LCB) between nucleotide sequences, with the level of similarity indicated by the height of the bars within each LCB. Genome alignments were performed using MAUVE.
