# Supplementary material for: Three novel bacteriophages isolated from the East African Rift Valley soda lakes
Source: Virol J. 2016 Dec 3;13:204. doi: 10.1186/s12985-016-0656-6 (PMC5135824; doi:10.1186/s12985-016-0656-6)
Supplement: Additional file 2: Table S1. — Predicted open reading frames on Shpa and closest BLASTp hit on the NCBI database. (DOCX 20 kb) [file 12985_2016_656_MOESM2_ESM.docx]

**Table S1.** Predicted open reading frames on Shpa and closest BLASTp hit on the NCBI database

| **ORF number** | **Size in amino acids** | **Start and end positions bp** | **Selected BLAST hits and comments; accession number; (length of protein on database in aa)** | **% Identity/Similarity (over number of aa)** |
| --- | --- | --- | --- | --- |
| 1 | 189 | 138-707 | HNH endonuclease *Paracoccus* sp. TRP WP_010400661.1 (99) / HNHc domain | 61/74 (57/93) |
| 2 | 169 | 499-1008 | Hypothetical protein *Sulfitobacter mediterraneus* WP_025049168.1 (115) /  Phage terminase small subunit *Burkholderia gladioli* WP_013696940.1 (140) | 52/70 (49/67)  35/47 (35/48) |
| 3 | 585 | 992-2746 | Terminase *Oceanibulbus* sp. HI0023 KZY05261.1 (604) | 77/85 (458/513) |
| 4 | 415 | 2748-3962 | Phage portal protein *Phaeobacter inhibens* DSM 17395 YP_006573648.1 (403) / COG4695 domain | 60/76 (243/309) |
| 5 | 218 | 3949-4602 | Putative prohead protease GP4 *Sinorhizobium fredii* HH103 YP_005188984.1 (222) / Peptidase_U35 domain | 53/69 (116/153) |
| 6 | 402 | 4634-5839 | Major capsid protein Gp5; Head protein *Sinorhizobium fredii* HH103 YP_005188983.1 (459) / Phage_Capsid domain | 66/79 (256/310) |
| 7 | 55 | 5898-6065 | Hypothetical protein *Rhizobium grahamii* WP_016553089.1 (60) | 58/67 (30/35) |
| 8 | 158 | 6062-6535 | DNA packaging protein *Paracoccus aminophilus* JCM 7686 YP_008404111.1 (168) / gp6 domain | 44/59 (70/95) |
| 9 | 110 | 6532-6861 | Phage head-tail adaptor, putative *Rhodobacter sphaeroides* ATCC 17029 YP_001045189.1 (111) / Phage_H_T_join domain | 54/68 (57/73) |
| 10 | 158 | 6947-7588 | Hypothetical protein *Rhodobacter* sp. AKP1 WP_009567283.1 (160) / phge_HK97_gp10 domain | 65/82 (88/112) |
| 11 | 125 | 7588-7962 | Hypothetical protein Rsph17029_3324 *Rhodobacter sphaeroides* ATCC 17029 YP_001045192.1 (126) / DUF3168 domain | 55/70 (68/87) |
| 12 | 145 | 7959-8393 | Hypothetical protein *Loktanella cinnabarina* WP_021693595.1 (142) /  Outer capsid protein *Paracoccus aminophilus* JCM 7686 YP_008403663.1 (146) / Phage_tail_3 domain | 61/75 (81/100)  50/70 (70/98) |
| 13 | 113 | 8393-8734 | Hypothetical protein DL1_11950 *Thioclava dalianensis* KEP68435.1 (114) | 31/48 (36/57) |
| 14 | 92 | 8749-9024 | Hypothetical protein *Loktanella cinnabarina* WP_021693597.1 (95) | 51/67 (47/63) |
| 15 | 705 | 9021-11135 | Hypothetical protein Sinme_2302 *Sinorhizobium meliloti* AK83 YP_004549635.1 (645) /  Phage tail tape measure protein *Phaeobacter inhibens* DSM 17395 YP_006573240.1 (831) | 34/50 (227/328)  31/47 (154/239) |
| 16 | 208 | 11135-11758 | Hypothetical protein EIO_1236 *Ketogulonicigenium vulgare* Y25 YP_003963679.1 (218) | 38/56 (82/123) |
| 17 | 220 | 11762-12421 | Hypothetical protein Rsph17025_1313 *Rhodobacter sphaeroides* ATCC 17025 YP_001167519.1 (218) | 44/59 (83/111) |
| 18 | 135 | 12418-12822 | Hypothetical protein EMVG_00018 *Emiliania huxleyi* virus PS401 AET73304.1 (137) | 58/70 (77/94) |
| 19 | 1073 | 12822-16040 | Hypothetical protein Rsph17025_1315 *Rhodobacter sphaeroides* ATCC 17025 YP_001167521.1 (1221) /  Tail protein *Nitratireductor indicus* WP_009756106.1 (917) / Phage-tail_3 domain | 43/56 (302/401)  40/57 (252/357) |
| 20 | 138 | 16333-16749 | Hypothetical protein, partial *Pseudomonas sp*. TJI-51 WP_009685311.1 (1982) | 32/47 (47/69) |
| 21 | 182 | 16746-17294 | Hypothetical protein *Paracoccus sp*. N5 WP_017999932.1 (179) | 33/51 (54/85) |
| 22 | 275 | 17878-18702 | Hypothetical protein *Paracoccus aminophilus* WP_020951092.1 (254) | 30/43 (41/59) |
| 23 | 131 | 18702-19097 | Hypothetical protein *Paracoccus aminophilus* WP_020951099.1 (113) | 36/48 (47/62) |
| 24 | 98 | 19491-19784 | Hypothetical protein *Ruegeria sp.* TM1040 WP_011538169.1 (98) | 34/59 (32/56) |
| 25 | 140 | 19787-20206 | Hypothetical protein *Dinoroseobacter shibae* WP_012178426.1 (138) | 39/59 (52/79) |
| 26 | 70 | 20203-20412 | Hypothetical protein *Paracoccus sp*. J55 WP_028712056.1 (66) | 40/64 (25/40) |
| 27 | 174 | 20473-20994 | Lysozyme *Brevundimonas diminuta* WP_003165060.1 (152) / PGRP domain | 51/64 (75/96) |
| 28 | 139 | 20996-21415 | Hypothetical protein *Roseobacter sp.* SK209-2-6 WP_008203963.1 (120) | 40/56 (42/59) |
| 29 | 187 | 22044-21481 | Hypothetical protein *Sphingobium yanoikuyae* WP_010337824.1 (192) | 44/54 (49/61) |
| 30 | 227 | 22721-22041 | Hypothetical protein *Nitratireductor indicus* WP_009756129.1 (231) | 74/84 (165/188) |
| 31 | 101 | 23014-22709 | Hypothetical protein *Pelagibaca bermudensis* WP_007799748.1  (100) | 33/54 (23/38) |
| 32 | 229 | 23700-23011 | Hypothetical protein *Rhizobium grahamii* WP_016556527.1 (240) | 40/54 (104/140) |
| 33 | 273 | 24515-23697 | Conserved hypothetical protein *Ruegeria lacuscaerulensis* WP_005980861.1 (285) / ParBc domain | 43/63 (83/123) |
| 34 | 129 | 25001-24615 | Restriction endonuclease *Brevundimonas diminuta* WP_003164135.1 (136) / McrA and HNHc domains | 65/79 (67/82) |
| 35 | 167 | 25498-24998 | Hypothetical protein TM1040_0782 *Ruegeria* sp. TM104 YP_612777.1 (167) | 65/80 (102/126) |
| 36 | 65 | 25695-25498 | Hypothetical protein *Dermabacter sp.* HFH0086 WP_016664899.1 (102) | 56/70 (15/19) |
| 37 | 84 | 25943-25692 | Hypothetical protein *Sinorhizobium meliloti* WP_017272340.1 (91) | 40/52 (36/48) |
| 38 | 146 | 26395-25958 | Single-stranded DNA-binding protein *Ruegeria lacuscaerulensis* WP_005983638.1 (161) / SSB_OBF domain | 67/74 (108/120) |
| 39 | 209 | 27018-26392 | Hypothetical protein *Pseudaminobacter salicylatoxidans* WP_019170719.1 (208) /  Phage-type endonuclease *Hyphomicrobium denitrificans* ATCC 51888 YP_003754272.1 (214) / YqaJ domain | 61/76 (126/160)  62/76 (126/155) |
| 40 | 244 | 27755-27024 | Hypothetical protein TM1040_0784 *Ruegeria* sp. TM1040 YP_612779.1 (240) /  ERF family protein *Methylobacterium nodulans* ORS 2060 YP_002499027.1 (244) / ERF domain | 43/62 (88/126)  37/56 (87/135) |
| 41 | 93 | 28681-28953 | Hypothetical protein *Pseudovibrio sp*. FO-BEG1e WP_014285584.1 (82) | 43/60 (25/35) |
| 42 | 119 | 28950-29303 | Hypothetical protein *Paracoccus* sp. TRP WP_010400599.1 (119) / DUF1064 domain | 67/79 (74/87) |
| 43 | 313 | 29309-30241 | Hypothetical protein *Paracoccus sp.* TRP WP_010400600.1 (316) / ParD domain | 80/88 (97/107) |
| 44 | 84 | 30238-30492 | Hypothetical protein *Paracoccus sp.* TRP WP_010400601.1 ( 83) /  Leucine-responsive transcriptional regulator *Rheinheimera baltica* WP_027670164.1 (162) / PRK 10533 domain | 64/75 (52/61)  28/50 (25/45) |
| 45 | 542 | 30486-32051 | DEAD/DEAH box helicase *Roseobacter* sp. AzwK-3b WP_007815863.1 (525) / DEXDc and HELICc domains | 66/80 (339/411) |
| 46 | 306 | 32048-32965 | Hypothetical protein, partial *Paracoccus* sp. TRP WP_010400962.1 (200) /  Primase 2 *Delftia acidovorans* SPH-1 YP_001562653.1 (309) / TOPRIM superfamily domain | 63/73 (126/147)  33/45 (74/103) |
| 47 | 59 | 33048-33227 | Hypothetical protein | N/A |
| 48 | 58 | 33267-33443 | Eyes absent *Cladonema radiatum* BAJ22814.1 (723) / GTPase_YqeH domain | 41/54 (19/25) |
| 49 | 178 | 33440-33976 | Hypothetical protein *Paracoccus sp.* TRP WP_010400356.1 (129) | 50/71 (23/33) |
| 50 | 352 | 34393-35448 | Hypothetical protein *Alistipes putredinis* WP_004327624.1 (541) | 48/62 (120/156) |
| 51 | 123 | 35453-35824 | Hypothetical protein *Clostridium papyrosolvens* WP_004616353.1 (103) / MnmC_Cterm and NAD_binding_8 superfamily domains | 40/57 (34/48) |
| 52 | 137 | 35821-36234 | Hypothetical protein EMVG_00054 *Emiliania huxleyi* virus PS401 AET73340.1 (163) / IENR1 domain | 31/44 (40/57) |
| 53 | 83 | 36231-36482 | Hypothetical protein BAMF_0715 *Bacillus amyloliquefaciens* DSM 7 YP_003919311.1 (123) | 30/50 (18/30) |
| 54 | 66 | 36479-36679 | Hypothetical protein TDEL_0G02300 *Torulaspora delbrueckii* XP_003682808.1 (506) | 46/75 (13/21) |
| 55 | 276 | 36703-37506 | Hypothetical protein | N/A |
| 56 | 191 | 37545-38117 | Hypothetical protein RSP_6190 *Rhodobacter sphaeroides* 2.4.1 YP_355122.1 (206) | 33/47 (67/98) |
