# Supplementary material for: Three novel bacteriophages isolated from the East African Rift Valley soda lakes
Source: Virol J. 2016 Dec 3;13:204. doi: 10.1186/s12985-016-0656-6 (PMC5135824; doi:10.1186/s12985-016-0656-6)
Supplement: Additional file 3: Table S2. — Predicted open reading frames on Mgbh1 and closest BLASTp hit on the NCBI database. (DOCX 23 kb) [file 12985_2016_656_MOESM3_ESM.docx]

**Table S2.** Predicted open reading frames on Mgbh1 and closest BLASTp hit on the NCBI database

| **ORF number** | **Size in amino acids** | **Start and end positions bp** | **Selected BLAST hits and comments; accession number; (length of protein on database in aa)** | **% Identity/Similarity (over number of aa)** |
| --- | --- | --- | --- | --- |
| 1 | 81 | 1149-1394 | TetR family transcriptional regulator *Thermomonospora curvata* DSM 43183 YP_003300163.1 (248) /  Head-tail joining protein *Vibrio alginolyticus* WP_005376955.1 (69) | 36/52 (28/41)  25/60 (15/37) |
| 2 | 64 | 1391-1585 | Hypothetical protein V074_02715 *Staphylococcus aureus* 2010-60-1240-1 EZV56794.1 (66) | 38/71 (20/37) |
| 3 | 115 | 1607-1954 | Hypothetical protein *Azospira oryzae* WP_014237633.1 (120) / MdlB domain | 34/56 (36/60) |
| 4 | 180 | 2008-2547 | Conserved hypothetical protein *Clostridium difficile* WP_022620775.1 (181) /  Antirestriction family protein *Clostridium difficile* WP_021425101.1 (237) / ArdA superfamily domain | 38/61 (39/63)  47/68 (36/53) |
| 5 | 258 | 2561-3334 | Hypothetical protein *Bacillus* phage vB_BanS-Tsamsa YP_008873260.1 (166) | 41/60 (60/89) |
| 6 | 82 | 3327-3572 | Hypothetical protein *Bacillus thuringiensis* WP_021728154.1 (83) | 48/63 (38/51) |
| 7 | 108 | 3686-4012 | Hypothetical protein *Gillisia marina* WP_010229014.1 (81) | 35/56 (25/41) |
| 8 | 58 | 4045-4218 | Phosphate acetyltransferase *Rhodoferax ferrireducens* WP_011462592.1 (683) | 40/50 (19/24) |
| 9 | 82 | 4218-4466 | Aminoglycoside phosphotransferase *Longispora albida* WP_026212065.1 (325) | 32/56 (23/41) |
| 10 | 233 | 4482-5180 | Hypothetical protein *Clostridium asparagiforme* WP_007718695.1 (230) | 32/50 (77/119) |
| 11 | 87 | 5301-5564 | Hypothetical protein ERIC1_2c00130 *Paenibacillus larvae subsp. larvae* DSM 25719 ETK25825.1 (269) | 43/53 (33/41) |
| 12 | 66 | 5566-5766 | Hypothetical protein *Ruminococcus gnavus* WP_024854954.1 (59) | 57/68 (20/24) |
| 13 | 146 | 7426-7854 | Phage protein *Bacillus amyloliquefaciens* DSM 7 YP_003919535.1 (143)/ HTH_Tnp_1_2 domain | 37/59 (47/75) |
| 14 | 571 | 7847-9562 | Hypothetical protein *Bacillus cereus* WP_000323342.1 (571) / psiM2_ORF9 and Terminase_6 domain | 53/72 (296/402) |
| 15 | 524 | 9578-11152 | Hypothetical protein *Bacillus cereus* WP_016123157.1 (513) /  Phage portal SPP1 Gp6-like family protein *Clostridium difficile* WP_021372446.1 (508) /  phage_prot_Gp6 domain | 48/66 (243/339)  25/45 (120/219) |
| 16 | 202 | 11218-11829 | Hypothetical protein *Bacillus* sp. UNC437CL72CviS29 WP_026593007.1 (192) /  Phage capsid protein *Staphylococcus aureus* WP_000392150.1 (206) | 30/59 (43/85)  39/57 (32/47) |
| 17 | 134 | 11842-12246 | Hypothetical protein *Bacillus cereus* WP_016123155.1 (128) | 67/78 (88/103) |
| 18 | 337 | 12294-13307 | Hypothetical protein *Bacillus cereus* WP_016123154.1 (342) /  Phage major capsid protein E *Brevibacillus* sp. CF112 WP_007779930.1 (334) / Phage_cap_E domain | 62/76 (211/260)  30/48 (100/161) |
| 19 | 70 | 13307-13519 | Hypothetical protein *Bacillus sp*. UNC437CL72CviS29 WP_026593004.1 (68) | 42/64 (26/40) |
| 20 | 113 | 13542-13883 | Hypothetical protein *Bacillus cereus* WP_000179866.1 (118) | 36/52 (40/58) |
| 21 | 256 | 13888-14658 | Phage protein *Bacillus sonorensis* WP_006636953.1 (269) | 31/50 (74/119) |
| 22 | 143 | 14659-15090 | Hypothetical protein *Bacillus sp*. CPSM8 WP_023856035.1 (129) / manB domain | 34/55 (48/78) |
| 23 | 122 | 15090-15458 | Hypothetical protein *Bacillus cereus* WP_000868438.1 (119) / HK97-gp10_like domain | 36/57 (27/43) |
| 24 | 148 | 15455-15901 | Hypothetical protein *Bacillus licheniformis* WP_025811921.1 (145) | 27/47 (37/66) |
| 25 | 173 | 15898-16419 | Tail protein *Anaerococcus* sp. PH9 WP_019116446.1 (174) / Phage_tail_2 superfamily domain | 43/60 (68/96) |
| 26 | 127 | 16527-16910 | Phage protein *Staphylococcus lugdunensis* N920143 YP_005759995.1 (119) /  Tail assembly protein *Staphylococcus* phage StB12 AFD22269.1 (121) / DUF3647 domain | 37/50 (42/58)  33/48 (42/62) |
| 27^a^ | 154 | 16820-17284 | Hypothetical protein *Streptococcus dysgalactiae* WP_003052379.1 (109) | 49/65 (43/58) |
| 28 | 1363 | 17288-21379 | Hypothetical protein *Staphylococcus intermedius* WP_019168219.1 (1642) / Phage tail tape measure  protein, TP901 family *Streptococcus ictaluri* WP_008089847.1 (1002) / tape_meas_TP901 and COG5412 domains | 31/52 (276/459)  29/47 (280/456) |
| 29 | 503 | 21391-22902 | Hypothetical protein *Virgibacillus halodenitrificans* WP_019377665.1 (478) /  Phage tail protein *Amphibacillus xylanus* NBRC 15112 YP_006844117.1 (477) / Sipho_tail domain | 44/61 (210/296)  36/58 (161/265) |
| 30 | 1286 | 22899-26759 | Hypothetical protein *Amphibacillus jilinensis* WP_017470613.1 (1354) / Prophage_tail superfamily domain | 39/56 (376/537) |
| 31 | 72 | 26752-26970 | Hypothetical protein BCBBV1cgp55 *Bacillus* phage BCJA1c YP_164433.1 (81) | 47/66 (32/45) |
| 32 | 47 | 26967-27110 | Protein of unknown function *Leuconostoc citreum* WP_004907716.1 (46) | 51/65 (22/28) |
| 33 | 93 | 27131-27412 | Bacteriophage holin-like protein (*bhlA*) *Bacillus oceanisediminis*  WP_019381952.1 (91) / DUF2762 domain | 62/78 (52/66) |
| 34 | 93 | 27409-27690 | Holin phage phi LC3 family *Firmicutes* bacterium CAG:41 WP_022229159.1 (92) / Phage_holin_1 domain | 60/81 (54/73) |
| 35 | 310 | 27690-28622 | Hypothetical protein JCM9140_3236 *Bacillus wakoensis* JCM 9140 GAE27119.1 (307) /  N-acetylmuramoyl-L-alanine amidase *Bacillus halodurans* C-125 NP_241832.1 (338) / PGRP and  PG_binding_1 domains | 55/69 (174/219)  63/76 (95/116) |
| 36 | 152 | 28656-29114 | Hypothetical protein *Brevibacillus agri* WP_005834793.1 (159) / cas_Csd_1 and HTH_17 domains | 39/61 (57/89) |
| 37 | 318 | 29625-30581 | Hypothetical protein *Gracilibacillus lacisalsi* WP_018933300.1 (323) /  Tyrosine recombinase XerD *Bacillus licheniformis* WP_016885269.1 (319) / DNA_BRE_C superfamily domain | 51/68 (164/218)  50/68 (160/219) |
| 38 | 122 | 30768-31136 | Transcriptional regulator *Bacillus cereus* WP_000108901.1 (145) / HTH_36 domain | 60/77 (72/93) |
| 39 | 85 | 31361-31618 | Hypothetical protein *Brevibacillus* sp. PhR WP_019123794.1 (84) / PRK15032 domain | 52/78 (43/65) |
| 40 | 127 | 31631-32015 | Hypothetical protein *Bacillus sonorensis* WP_006636892.1 (124) | 46/64 (49/69) |
| 41 | 100 | 32032-32334 | Hypothetical protein GBK2_48 *Geobacillus* phage GBK2 YP_009010519.1 (146) / IDEAL domain | 38/61 (18/29) |
| 42 | 128 | 32544-32930 | Hypothetical protein *Bacillus pumilus* WP_003213512.1 (113) | 44/59 (51/70) |
| 43 | 97 | 32930-33223 | Hypothetical protein *Brevibacillus brevis* WP_017248621.1 (150) | 51/66 (38/49) |
| 44^a^ | 118 | 33239-33595 | Hypothetical protein *Brevibacillus borstelensis* WP_003386601.1 (117) | 69/84 (18/22) |
| 45 | 62 | 33610-33798 | Hypothetical protein Z962_p0049 *Clostridium botulinum* C/D str. BKT12695 KEH91673.1 (99) | 35/58 (16/27) |
| 46 | 63 | 33837-34028 | Hypothetical protein *Bacillus licheniformis* WP_016885320.1 (74) | 69/83 (43/52) |
| 47 | 239 | 34340-35059 | Hypothetical protein *Bacillus licheniformis* WP_017474270.1 (255) /  DNA replication protein *Paenibacillus* sp. FSL R7-269 ETT40925.1 (259) / AAA domain | 46/63 (119/163)  43/62 (110/159) |
| 48 | 459 | 35056-36435 | DNA helicase *Bacillus cereus* WP_001106133.1 (447) / RecA-like_NTPases superfamily domain | 49/67 (220/305) |
| 49 | 316 | 36746-37696 | DNA primase *Bacillus amyloliquefaciens* DSM 7 YP_003919491.1 (331) / TOPRIM_DnaG_primases domain | 43/57 (139/186) |
| 50 | 99 | 37674-37973 | Hypothetical protein *Bacillus* cereus WP_000448739.1 (112) | 33/52 (23/37) |
| 51 | 45 | 38052-38189 | Hypothetical protein *Escherichia coli* WP_000651011.1 (60) | 46/65 (168/23) |
| 52 | 163 | 38378-38869 | Hypothetical protein *Bacillus licheniformis* WP_017474278.1 (204) | 52/63 (51/62) |
| 53 | 433 | 39451-40752 | Transposase *Bacillus cereus* WP_002073819.1 (453) / tspaseT_teng_C and OrfB_IS605 domains | 38/58 (164/252) |
| 54 | 264 | 41046-41840 | Hypothetical protein *Brevibacillus laterosporus* WP_018672625.1 (253) / Single-stranded DNA-binding protein *Bacillus cereus* WP_000512818.1 (250) | 57/70 (138/172)  50/63 (125/159) |
| 55 | 128 | 41852-42238 | Hypothetical protein *Brevibacillus borstelensis* WP_003386573.1 (135) | 40/59 (52/77) |
| 56 | 140 | 42258-42680 | Hypothetical protein *Bacillus cereus* WP_016092017.1 (138) | 43/60 (60/85) |
| 57 | 717 | 42694-44847 | Hypothetical protein *Bacillus cereus* WP_002098473.1 (727) /  DNA-directed DNA polymerase *Brevibacillus borstelensis* WP_003386571.1 (730) / DnaQ_like_exo superfamily and DNA_pol_A_pol_I_C domains | 54/71 (387/515)  55/70 (379/486) |
| 58 | 302 | 44941-45849 | Hypothetical protein *Paenibacillus elgii* WP_010495009.1 (738) | 32/43 (78/107) |
| 59 | 373 | 45851-46972 | Hypothetical protein *Paenibacillus terrigena* WP_018755163.1 (472) | 35/50 (109/158) |
| 60 | 328 | 47008-47994 | Hypothetical protein *Bacillus cereus* WP_002203567.1 (364) | 48/67 (156/220) |
| 61 | 176 | 48185-48715 | Crossover junction endodeoxyribonuclease RuvC *Bacillus cereus* WP_001254323.1 (164) / RuvC_resolvase superfamily domain | 31/49 (53/86) |
| 62 | 118 | 48908-49264 | Ribonucleotide reductase *Bacillus* sp. CPSM8 WP_023856071.1 (118) / nrdI domain | 56/71 (66/84) |
| 63 | 681 | 49290-51335 | Ribonucleotide-diphosphate reductase subunit alpha *Bacillus licheniformis* WP_016885228.1 (699) / RNR_N and RNR_I domains | 79/88 (549/611) |
| 64 | 329 | 51348-52337 | Ribonucleotide-diphosphate reductase *Bacillus* WP_008344010.1 (326) / RNRR2 domain | 72/84 (238/277) |
| 65 | 57 | 52337-52510 | Hypothetical protein | N/A |
| 66 | 123 | 52586-52957 | Hypothetical protein MCCL_0956 *Macrococcus caseolyticus* JCSC5402 YP_002560359.1 (100) /  ArpR *Virgibacillus* sp. CM-4 WP_021292107.1 (101) / NTP-PPase_u3 domain | 68/87 (65/83)  62/79 (62/79) |
| 67 | 138 | 53093-53509 | TPA_inf: HDC06314 Drosophila melanogaster DAA02496.1 (116) | 27/58 (15/32) |
| 68 | 65 | 53502-53699 | Type IIA topoisomerase, B subunit *Solibacillus silvestris* StLB046 YP_006461973.1 (70) | 65/80 (39/48) |
| 69 | 80 | 53700-53942 | Hypothetical protein BcerKBAB4_5285 *Bacillus weihenstephanensis* KBAB4 YP_001642755.1 (139) | 43/59 (34/47) |
| 70 | 256 | 53943-54713 | Thymidylate synthase *Bacillus pumilus* WP_003213632.1 (262) / Thy 1 superfamily domain | 68/77 (175/201) |
| 71 | 52 | 54751-54909 | Hypothetical protein | N/A |
| 72 | 86 | 54948-55208 | AMP-binding enzyme family protein *Mycobacterium ulcerans* str. Harvey EUA88506.1 (419) / PLN02447 domain | 39/54 (22/31) |
| 73 | 70 | 55357-55569 | Flavin reductase *Mesorhizobium sp*. LSJC280B00 WP_023674995.1 (599) | 45/66 (19/28) |
| 74 | 68 | 55583-55789 | Molecular chaperone, small heat shock protein halophilic archaeon J07HX64 WP_021041678.1 (117) | 34/57 (21/35) |
| 75 | 93 | 55809-56090 | Hypothetical protein *Bacillus cereus* WP_000873127.1 (94) | 41/59 (38/55) |
| 76 | 146 | 56188-56628 | SPBc2 prophage-derived protein YorM *Bacillus sonorensis* WP_006636923.1 (196) / COG3584 domain | 60/78 (63/82) |
| 77 | 300 | 56649-57551 | Hypothetical protein SSIL_1402 *Solibacillus silvestris* StLB046 YP_006461971.1 (90) | 57/75 (44/58) |
| 78 | 114 | 57548-57892 | Putative alcohol dehydrogenase *Bacillus sp*. TS-2 GAF66035.1 (107) | 33/47 (19/27) |
| 79 | 111 | 57987-58322 | Hypothetical protein *Paenibacillus terrigena* WP_018755180.1 (131) / COG3413 domain | 33/49 (33/50) |
| 80 | 152 | 58319-58777 | Hypothetical protein *Brevibacillus borstelensis* WP_003386546.1 (174) | 31/50 (38/62) |

^a^ Possible read through translation
