# Supplementary material for: Three novel bacteriophages isolated from the East African Rift Valley soda lakes
Source: Virol J. 2016 Dec 3;13:204. doi: 10.1186/s12985-016-0656-6 (PMC5135824; doi:10.1186/s12985-016-0656-6)
Supplement: Additional file 4: Table S3. — Predicted open reading frames on Shbh1 and closest BLASTp hit on the NCBI database. (DOCX 37 kb) [file 12985_2016_656_MOESM4_ESM.docx]

**Table S3.** Predicted open reading frames on Shbh1 and closest BLASTp hit on the NCBI database

| **ORF number** | **Size in amino acids** | **Start and end positions (bp)** | **Selected BLAST hits and comments; accession number; (length of protein on database in aa)** | **% Identity/Similarity (over number of aa)** |
| --- | --- | --- | --- | --- |
| 1 | 243 | 477-1208 | Hypothetical protein Grass_157 *Bacillus* phage Grass YP_008771523.1 (246) / PRK08626 flavoprotein domain | 70/83 (171/204) |
| 2 | 169 | 1223-1732 | Hypothetical protein Grass_158 *Bacillus* phage Grass YP_008771524.1 (158) | 59/78 (91/121) |
| 3 | 231 | 1791-2483 | Hypothetical protein Bcp1_187 *Bacillus* phage Bcp1 YP_009031469.1 (202) /  Putative DNA binding protein *Bacillus* phage phiNIT1 YP_008318415.1 (186) | 39/62 (52/85)  33/47 (52/75) |
| 4 | 205 | 3108-3725 | Hypothetical protein *Bacillus* phage phiNIT1 YP_008318418.1 (247) | 32/52 (76/124) |
| 5 | 128 | 3826-4212 | Flagellar basal-body rod modification protein FlgD *Thalassobacter arenae* WP_021102414.1 (216) | 25/44 (26/46) |
| 6 | 62 | 4327-4515 | Hypothetical protein *Brevibacillus brevis* WP_017248790.1 (64) | 57/83 (34/50) |
| 7 | 151 | 4932-5387 | Hypothetical protein *Paenibacillus polymyxa* WP_013385909.1 (84) | 40/60 (27/41) |
| 8 | 71 | 5448-5663 | Hypothetical protein *Paenibacillus taiwanensis* WP_028547214.1 (76) / hemeryth_dom domain | 38/63 (22/37) |
| 9 | 81 | 5679-5921 | Hypothetical protein BTS2_3322 *Bacillus* sp. TS-2 GAF66421.1 (84) | 62/75 (48/59) |
| 10 | 73 | 6622-6843 | Hypothetical protein PBF_21998 *Bacillus firmus* DS1 EWG08882.1 (70) | 34/61 (21/38) |
| 11 | 45 | 6874-7011 | Hypothetical protein *Exiguobacterium sp.* NG55 WP_031422968.1 (175) | 43/67 (20/31) |
| 12 | 139 | 7176-7595 | Hypothetical protein LL3_02287 *Bacillus amyloliquefaciens* YP_005546051.1 LL3 (120) | 55/63 (77/88) |
| 13 | 201 | 7675-8280 | Metallophosphoesterase *Bacillus* phage Troll YP_008430899.1 (188) / MPP_AQ1575 domain | 45/64 (75/107) |
| 14 | 113 | 8280-8621 | Hypothetical protein TREMEDRAFT_58785 *Tremella mesenterica* DSM 1558 XP_007001401.1 (1048) / PRK00451 domain | 30/56 (29/55) |
| 15 | 168 | 8618-9124 | Trans membrane emp24 domain containing protein 7 *Echinococcus granulosus* CDJ24841.1 (234) | 31/50 (27/44) |
| 16 | 218 | 9117-9773 | Hypothetical protein BN990_04251 *Virgibacillus* sp. Vm-5 CDQ41872.1 (214) | 26/48 (57/104) |
| 17 | 106 | 9770-10090 | Uncharacterized protein C20orf24 homolog *Ciona intestinalis* XP_002120089.1 (112) | 32/52 (28/46) |
| 18 | 361 | 10269-11354 | Hypothetical protein SIOphi_00635 *Bacillus* phage SIOphi AGK86935.1 (361) /  Plasmid segregation protein *Bacillus* phage Grass YP_008771547.1 (359) / ParM_like domain | 74/86 (263/309)  69/84 (244/300) |
| 19 | 112 | 11338-11676 | Hypothetical protein Grass_182 *Bacillus* phage Grass YP_008771548.1 (102) | 47/62 (51/67) |
| 20 | 204 | 11806-12420 | Metalloendopeptidase-like membrane protein *Bacillus* phage SIOphi AGK86933.1 (189) / Peptidase_M23 domain | 54/71 (102/135) |
| 21 | 66 | 12564-12764 | Hypothetical protein *Bacillus subtilis* WP_017696845.1 (66) / oxa_formate Anti domain | 37/65 (25/44) |
| 22 | 195 | 12844-13431 | Hypothetical protein *Bacillus* phage Bobb AII27970.1 (182) | 49/68 (96/134) |
| 23 | 350 | 13542-14594 | Hypothetical protein *Bacillus* phage phiNIT1 YP_008318435.1 (395) / Branch_AA_trans domain | 56/73 (220/293) |
| 24 | 42 | 14615-14743 | Hypothetical protein Grass_186 *Bacillus* phage Grass YP_008771552.1 (39) | 71/86 (27/33) |
| 25 | 768 | 14727-17033 | DNA translocase *Bacillus* phage SIOphi AGK86930.1 (737) / FtsK_SpoIIIE domain | 55/71 (419/543) |
| 26 | 210 | 17165-17797 | Heat shock protein ClpB-like protein *Roseburia sp*. CAG:303 WP_022449394.1 (860) | 31/52 (32/54) |
| 27 | 80 | 17799-18041 | Hypothetical protein *Bacillus azotoformans* WP_003330919.1 (79) | 31/58 (22/41) |
| 28 | 133 | 18051-18452 | Hypothetical protein *Lactococcus lactis* WP_014570740.1 (40) | 31/68 (11/24) |
| 29 | 272 | 18442-19260 | Inner centromere protein A-like *Aplysia californica* XP_005108347.1 (1227) | 24/40 (55/92) |
| 30 | 213 | 19360-20001 | Hypothetical protein *Bacillus atrophaeus* WP_010787769.1 (193) | 53/74 (70/98) |
| 31 | 87 | 19988-20251 | DNA primase *Lactobacillus curvatus* WP_004270801.1 (626) | 33/60 (15/27) |
| 32 | 99 | 20291-20569 | Hypothetical protein JCM16418_5116 *Paenibacillus pini* JCM 16418 GAF10885.1 (79) | 41/52 (21/27) |
| 33^a^ | 167 | 20610-21116 | Arginine--tRNA ligase *Staphylococcus sp*. CAG:324 WP_022291694.1 (562) / Imm_39 domain | 29/48 (38/64) |
| 34 | 95 | 21131-21418 | Hypothetical protein *Bacillus* phage phiNIT1 YP_008318451.1 (92) | 33/49 (31/47) |
| 35 | 98 | 21505-21801 | Hypothetical protein *Bacillus cereus* group WP_000978593.1 (101) | 52/70 (52/70) |
| 36 | 86 | 21956-22216 | Shikimate dehydrogenase *Ruminococcus* sp. SR1/5 WP_015527108.1 (288) | 36/50 (24/34) |
| 37 | 154 | 22397-22861 | Glutamyl-tRNA synthetase *Streptosporangium amethystogenes* WP_030913618.1 (443) | 27/42 (39/62) |
| 38 | 52 | 22865-23023 | ATP-dependent DNA helicase RecQ *Lactobacillus acidophilus* WP_013641835.1 (588) | 34/59 (16/28) |
| 39 | 154 | 23011-23475 | Glycerate dehydrogenase Oceanimonas sp. GK1 WP_014291584.1 (316) | 37/59 (18/29) |
| 40 | 84 | 23551-23805 | Hypothetical protein *Bacillus* phage phiNIT1 YP_008318462.1 (79) | 32/47 (24/35) |
| 41 | 76 | 23903-24133 | Hypothetical protein SIOphi_00505 *Bacillus* phage SIOphi AGK86909.1 (72) | 39/67 (28/48) |
| 42 | 180 | 24210-24752 | Hypothetical protein PPSC2_p0010 *Paenibacillus polymyxa* SC2 YP_003965473.1 (182) / Cas_VVA1548 CRISPR related domain | 61/75 (109/135) |
| 43 | 91 | 24754-25029 | Hypothetical protein *Verminephrobacter eiseniae* WP_011811856.1 (134) | 52/66 (17/22) |
| 44 | 117 | 25120-25470 | Uncharacterized protein CBO05P2_130 *Clostridium botulinum* B str. Osaka05 BAO05155.1 (190) | 50/63 (56/71) |
| 45 | 60 | 25643-25825 | Hypothetical protein *Nostoc sp.* PCC 7107 WP_015112564.1 (209) | 47/61 (17/22) |
| 46 | 95 | 25822-26109 | Hypothetical protein DAPPUDRAFT_114603 *Daphnia pulex* EFX68399.1 (264) | 33/47 (31/45) |
| 47 | 128 | 26125-26511 | Hydroxylamine reductase Firmicutes bacterium CAG:102 WP_016406711.1 (556) | 33/50 (27/41) |
| 48 | 175 | 26779-27320 | Hypothetical protein Nocardiopsis sp. CNT312 WP_028648444.1 (621) | 44/64 (20/29) |
| 49 | 91 | 28377-28652 | PTS sucrose transporter subunit IIABC *Robinsoniella sp*. KNHs210 WP_027296208.1 (646) | 31/55 (21/37) |
| 50 | 182 | 28738-29286 | Hypothetical protein *Bacillus* phage vB_BanS-Tsamsa YP_008873260.1 (166) / ocr domain anti-restriction prot | 42/63 (66/100) |
| 51 | 53 | 29492-29653 | Pogo transposable element, putative *Talaromyces marneffei* ATCC 18224 XP_002148149.1 (525) | 33/53 (17/28) |
| 52 | 74 | 29707-29931 | Hypothetical protein *Thioalkalivibrio thiocyanodenitrificans* WP_018234366.1 (71) | 41/64 (15/24) |
| 53 | 86 | 29954-30214 | Hypothetical protein EUGRSUZ_I00481 *Eucalyptus grandis* KCW54528.1 (969) | 33/56 (18/31) |
| 54 | 79 | 30211-30450 | Cytochrome P450 *Brevibacillus laterosporus* WP_031411899.1 (406) | 32/50 (22/34) |
| 55 | 111 | 30551-30886 | Capsid protein *Lysinibacillus sphaericus* WP_012295658.1 (485) | 23/45 (25/50) |
| 56 | 75 | 31482-31709 | Hypothetical protein CKL_3364 *Clostridium kluyveri* DSM 555 YP_001396738.1 (60) | 44/70 (27/43) |
| 57 | 118 | 31801-32157 | Hypothetical protein *Clostridiales* bacterium VE202-15 WP_024734696.1 (123) | 66/76 (80/93) |
| 58 | 193 | 32299-32880 | Hypothetical protein *Bacillus cereus* WP_002124315.1 (188) /  Exonuclease *Bacillus* phage Evoli AHZ09842.1 (194) / dexA exonuclease domain | 65/78 (123/149)  53/69 (100/131) |
| 59 | 52 | 33143-33301 | DNA/RNA helicase, SNF2 *Clostridium acetobutylicum* DSM 1731 YP_004635889.1 (54) | 57/78 (29/40) |
| 60 | 159 | 33327-33806 | Hypothetical protein BCP78_0127 *Bacillus* phage BCP78 YP_006907962.1 (142) | 76/88 (73/85) |
| 61 | 73 | 33819-34040 | Hypothetical protein | N/A |
| 62 | 94 | 34892-35176 | Hypothetical protein BAPNAU_1618 *Bacillus amyloliquefaciens* subsp. *plantarum* NAU-B3  YP_008626260.1 (86) / DUF4176 | 41/62 (35/53) |
| 63 | 53 | 35289-35450 | Hypothetical protein | N/A |
| 64 | 40 | 35540-35662 | Hypothetical protein | N/A |
| 65 | 80 | 35943-36185 | Hypothetical protein JCM16418_5154 *Paenibacillus pini* JCM 16418 GAF10917.1 (80) / DUF1247 domain | 64/78 (51/63) |
| 66 | 67 | 36227-36430 | Hypothetical protein GMDG_01909 *Pseudogymnoascus destructans* 20631-21 ELR05947.1 (1492) | 41/70 (15/26) |
| 67 | 130 | 36499-36891 | Phage protein *Paenibacillus sp.* FSL H8-237 ETT55207.1 (133) / YopX domain | 43/62 (55/80) |
| 68 | 256 | 37074-37844 | Transposase, IS605 OrfB family *Virgibacillus halodenitrificans* CDQ31559.1 (377) / OrfB_IS605 domain | 93/96 (235/245) |
| 69 | 82 | 37855-38013 | Transposase *Virgibacillus halodenitrificans* CDQ32446.1(132) / Y1_Tnp IS200 domain | 95/100 (76/80) |
| 70 | 119 | 38226-38585 | DNA repair protein recA homolog 2, mitochondrial-like *Brachypodium distachyon*  XP_003567210.1 (356) | 24/44 (29/54) |
| 71 | 91 | 38630-38905 | Hypothetical protein *Bacillus cereus* WP_001229813.1 (83) | 48/66 (41/57) |
| 72 | 87 | 38936-39199 | Hypothetical protein *Paenibacillus polymyxa* WP_025365314.1 (79) | 36/58 (31/50) |
| 73 | 54 | 39672-39836 | Hypothetical protein | N/A |
| 74 | 101 | 39858-40163 | Hypothetical protein Noc_1823 *Nitrosococcus oceani* ATCC 19707 YP_343821.1 (262) | 36/47 (20/26) |
| 75 | 60 | 40396-40578 | Hypothetical protein TROLL_146 *Bacillus* phage Troll YP_008430930.1 (62) | 45/73 (27/44) |
| 76 | 48 | 40593-40739 | Hypothetical protein *Bacillus sp*. WBUNB009 WP_017658281.1 (48) | 52/75 (25/36) |
| 77 | 321 | 40739-41704 | Methyltransferase *Pelosinus* WP_007932066.1 (366) /  DNA-cytosine methyltransferase, partial *Pelosinus fermentans WP_007943738.1* (299) / dcm domain | 46/57 (168/212)  49/62 (124/160) |
| 78 | 91 | 41724-41999 | Hypothetical protein *Bacillus cereus* WP_000424057.1 (94) | 51/70 (43/60) |
| 79 | 97 | 41992-42288 | Hypothetical protein *Halococcus salifodinae* WP_005042072.1 (69) | 41/74 (16/29) |
| 80 | 136 | 42763-43170 | Hypothetical protein *Bacillus cereus* WP_000445917.1 (143) | 54/70 (69/90) |
| 81 | 89 | 43172-43441 | Hypothetical protein PANG_00046 *PaeniBacillus* phage PG1 YP_008129910.1 (69) | 47/72 (32/49) |
| 82 | 163 | 43712-44203 | AbrB family transcriptional regulator *Peptococcaceae* bacterium SCADC1_2_3 KFD40645.1 (93) | 57/80 (17/24) |
| 83 | 98 | 44222-44518 | Hypothetical protein BTP1_49 *Bacillus* phage phiBTP1 AGM61415.1 (102) | 50/60 (51/61) |
| 84 | 95 | 44585-44872 | ATP-dependent exonuclease subunit A *Streptococcus pyogenes* NZ131 YP_002285621.1 (1222) | 33/51 (21/33) |
| 85 | 252 | 44923-45681 | Hypothetical protein *Bacillus* WP_009794093.1 (248) / DUF3603 domain | 33/55 (78/133) |
| 86 | 191 | 45784-46359 | Dephospho-CoA kinase *Bacillus* phage Bcp1 YP_009031325.1 (199) / PRK08356 Deoxynucleoside monophosphate kinase domain | 47/68 (90/130) |
| 87 | 292 | 46373-47251 | Thymidylate synthase *Bacillus* phage SIOphi AGK86851.1 (294) / TS_Pyrimidine_Hmase domain | 66/80 (193/235) |
| 88 | 246 | 47268-48008 | Putative nucleotidyltransferase *Bacillus azotoformans* MEV2011 KEF37533.1 (241) / COG3541 domain | 41/63 (100/154) |
| 89 | 69 | 48032-48241 | Hypothetical protein BCP78_0021 *Bacillus* phage BCP78 YP_006907856.1 (80) | 58/82 (39/55) |
| 90 | 191 | 48347-48922 | Hypothetical protein SIOphi_00205 *Bacillus* phage SIOphi AGK86849.1 (188) | 57/71 (105/132) |
| 91 | 475 | 48923-50350 | Hypothetical protein *Bacillus* phage vB_BceM-Bc431v3 YP_007676917.1 (470) / PcfJ and PI-PLCc_delta domains | 49/69 (236/333) |
| 92 | 289 | 50444-51313 | Hypothetical protein Bcp1_049 *Bacillus* phage Bcp1 YP_009031329.1 (253) / AAD_C domain | 21/37 (44/80) |
| 93 | 228 | 51350-52036 | PhoH family protein *Bacillus* phage SIOphi AGK86847.1 (229) / PhoH domain | 68/83 (156/191) |
| 94 | 311 | 52235-53170 | N-acetylmuramoyl-L-alanine amidase *Bacillus licheniformis* WP_026080833.1 (294) / MurNAc-LAA and PG_binding_1 domains | 43/62 (136/197) |
| 95 | 106 | 53216-53536 | Hypothetical protein SIOphi_00185 *Bacillus* phage SIOphi AGK86845.1 (106) | 56/70 (59/74) |
| 96 | 173 | 53523-54044 | Putative membrane protein *Bacillus* phage Bcp1 YP_009031332.1 (254) | 37/58 (93/146) |
| 97 | 296 | 54220-55110 | Hypothetical protein SIOphi_00175 *Bacillus* phage SIOphi AGK86843.1 (298) | 50/68 (145/198) |
| 98 | 600 | 55198-57000 | Terminase large subunit *Bacillus* phage Bcp1 YP_009031337.1 (598) / Terminase_GpA superfamily domain | 75/88 (450/530) |
| 99 | 140 | 57002-57424 | Hypothetical protein *Bacillus* phage vB_BceM-Bc431v3 YP_007676907.1 (137) | 65/81 (90/113) |
| 100 | 97 | 57411-57704 | Hypothetical protein SIOphi_00145 *Bacillus* phage SIOphi AGK86837.1 (96) / BcsB domain | 61/77 (54/69) |
| 101 | 176 | 57688-58218 | Hypothetical protein SIOphi_00140 *Bacillus* phage SIOphi AGK86836.1 (171) | 41/60 (72/106) |
| 102 | 88 | 58237-58503 | Putative membrane protein *Bacillus* phage vB_BceM-Bc431v3 YP_007676904.1 (93) | 29/61 (25/53) |
| 103 | 112 | 58900-59238 | Hypothetical protein *Bacillus* phage phiNIT1 YP_008318315.1 (120) | 56/74 (56/74) |
| 104 | 104 | 59264-59578 | Hypothetical protein SIOphi_00120 *Bacillus* phage SIOphi AGK86832.1 (107) / PHA02414 domain | 65/82 (65/82) |
| 105 | 75 | 59601-59828 | Hypothetical protein G380_gp205 *Bacillus* phage phiAGATE YP_007349211.1 (78) | 28/51 (17/31) |
| 106 | 80 | 59865-60107 | HTH binding domain protein *Bacillus* phage Troll YP_008430856.1 (95) / HTH_XRE domain | 69/83 (50/60) |
| 107 | 77 | 61306-61539 | Hypothetical protein *Halalkalibacillus halophilus* WP_027963760.1 (96) /  Putative XRE family transcriptional regulator *Bacillus* phage phiNIT1 YP_008318319.1 (89) / HTH_XRE domain | 62/79 (48/61)  52/75 (40/58) |
| 108 | 109 | 61822-62151 | Hypothetical protein Grass_66 *Bacillus* phage Grass YP_008771432.1 (109) | 57/78 (62/85) |
| 109 | 550 | 62170-63822 | Putative portal protein *Bacillus* phage phiNIT1 YP_008318325.1 (553) / Phage_portal domain | 74/86 (413/481) |
| 110 | 265 | 63983-64780 | Hypothetical protein SIOphi_00085 *Bacillus* phage SIOphi AGK86825.1 (279) / Putative prohead protease *Bacillus* phage BCP78 YP_006908058.1 (265) / Peptidase_U35 domain | 69-89 (176/227) |
| 111 | 257 | 64805-65578 | Hypothetical protein SIOphi_00080 *Bacillus* phage SIOphi AGK86824.1 (305) | 40/53 (70/94) |
| 112 | 474 | 65732-67156 | Major capsid protein *Bacillus* phage phiNIT1 YP_008318328.1 (481) | 76/85 (355/403) |
| 113 | 94 | 67270-67554 | Hypothetical protein SIOphi_00070 *Bacillus* phage SIOphi AGK86822.1 (82) | 58/72 (29/36) |
| 114 | 2910 | 67581-68453 | Hypothetical protein SIOphi_00065 *Bacillus* phage SIOphi AGK86821.1 (290) /  Putative structural protein *Bacillus* phage Bcp1 YP_009031357.1 (291) | 64/81 (180/229)  64/81 (181/229) |
| 115 | 281 | 68467-69312 | Hypothetical protein G380_gp157 *Bacillus* phage phiAGATE YP_007349196.1 (281) | 58/73 (150/191) |
| 116 | 209 | 69313-69942 | Hypothetical protein SIOphi_00055 *Bacillus* phage SIOphi AGK86819.1 (211) | 64/80 (130/164) |
| 117 | 303 | 69954-70865 | Hypothetical protein Grass_74 *Bacillus* phage Grass YP_008771440.1 (288) | 56/69 (162/200) |
| 118 | 568 | 71178-72884 | Hypothetical protein SIOphi_00040 *Bacillus* phage SIOphi AGK86816.1 (569) /  Tail sheath protein *Bacillus* phage Grass YP_008771442.1 (569) / Phage_sheath_1 domain | 70/85 (400/488)  69/84 (395/482) |
| 119 | 141 | 72934-73359 | Hypothetical protein *Bacillus* phage phiNIT1 YP_008318337.1 (141) /  Structural protein *Bacillus* phage B4 YP_006908487.1 (142) | 92/96 (130/136)  92/97 (129/137) |
| 120 | 96 | 73446-73736 | Group-specific protein *Bacillus bataviensis* WP_007085602.1 (113) / IDEAL domain | 34/56 (30/49) |
| 121 | 201 | 73846-74451 | Putative cell wall-binding, peptidase-related domain protein *Bacillus* phage phiAGATE YP_007349189.1 (202) / COG3584 domain | 59/74 (119/151) |
| 122 | 137 | 74575-74988 | Hypothetical protein G380_gp148 *Bacillus* phage phiAGATE YP_007349187.1 (135) | 60/79 (74/98) |
| 123 | 191 | 75056-75631 | Hypothetical protein *Bacillus* phage phiNIT1 YP_008318341.1 (208) / RRM2_hnRNPR_like domain | 64/79 (134/168) |
| 124 | 1240 | 75699-79421 | Peptidoglycan hydrolase *Bacillus* phage CAM003 YP_009036993.1 (1289) /  Tapemeasure *Bacillus* phage Troll YP_008430878.1 (1316) / Glucosaminidase domain | 41/58 (550782)  39/57 (524/779) |
| 125 | 730 | 79478-81670 | Tail lysin *Bacillus* phage Grass YP_008771449.1 (718) / NLPC_P60 domain | 67/81 (486/595) |
| 126 | 1511 | 81682-86217 | Putative tail fiber *Bacillus* phage Bcp1 YP_009031370.1 (678) /  Hypothetical protein BN982_04120 *Halobacillus dabanensis* CDQ21711.1 (2264) Fragmented, but no introns according to RNA weasel | 48/66 (286/395)  35/53 (195/294) |
| 127 | 104 | 86251-86565 | PREDICTED: putative germ cell-specific gene 1-like protein 2 *Cynoglossus semilaevis* XP_008327354.1 (303) | 24/44 (23/43) |
| 128 | 115 | 86612-86959 | PREDICTED: moesin/ezrin/radixin homolog 1-like isoform X1 *Apis dorsata* XP_006616909.1 (495) / PLN03223 domain | 37/81 (30/50) |
| 129 | 247 | 87031-87774 | Hypothetical protein SIOphi_01000 *Bacillus* phage SIOphi AGK87008.1 (255) | 63/75 (160/190) |
| 130 | 175 | 87774-88301 | Hypothetical protein *Bacillus* phage phiNIT1 YP_008318355.1 (172) | 55/73 (92/123) |
| 131 | 239 | 88294-89103 | Hypothetical protein SIOphi_00990 *Bacillus* phage SIOphi AGK87006.1 (246) /  Baseplate assembly protein *Bacillus* phage Grass YP_008771458.1 (245) / COG3628 domain | 65/83 (154/198)  63/81 (150/193) |
| 132 | 348 | 89028-90074 | Hypothetical protein SIOphi_00985 *Bacillus* phage SIOphi AGK87005.1 (348) /  Baseplate J family protein *Bacillus* phage BCP78 YP_006908028.1 (348)/ Baseplate_J domain | 65/83 (225/291)  60/79 (208/275) |
| 133 | 516 | 90091-91641 | Baseplate protein *Bacillus* phage Grass YP_008771460.1 (500) | 48/64 (231/310) |
| 134 | 176 | 91707-92237 | Hypothetical protein SIOphi_00970 *Bacillus* phage SIOphi AGK87002.1 (176) /  Structural protein *Enterococcus* phage phiEF24C YP_001504149.1 (181) | 57/73 (100/129)  47/63 (81/109) |
| 135 | 797 | 92266-94659 | YomR *Erwinia* phage PhiEaH1 YP_009010246.1 (867) / Contains repeated sequence | N/A |
| 136 | 1166 | 95056-98556 | Hypothetical protein G380_gp133 *Bacillus* phage phiAGATE YP_007349376.1 (1161) /  Putative tail protein *Bacillus* phage phiNIT1 YP_008318362.1 (1166) | 58/74 (676/871)  58/74 (684/872) |
| 137 | 949 | 98738-101587 | SPBc2 prophage-derived protein YomR *Bacillus amyloliquefaciens* SQR9 AHZ16169.1 (448) / 235kDa-fam domain / Contains repeated sequence | N/A |
| 138 | 100 | 101590-101892 | Hypothetical protein ECL_01718 *Enterobacter cloacae subsp. cloacae* ATCC 13047 YP_003612225.1 (91) | 32/53 (29/49) |
| 139 | 74 | 101893-102117 | Hypothetical protein ABC1345 *Bacillus clausii* KSM-K16 YP_174844.1 (126) | 40/63 (21/33) |
| 140 | 52 | 102119-102277 | Hypothetical protein ABC2819 *Bacillus clausii* KSM-K16 YP_176314.1 (59) / Phage_XkdX domain | 64/86 (32/43) |
| 141 | 590 | 102813-104585 | Recombination helicase *Bacillus* phage Grass YP_008771466.1 (590) / ResIII and HELICc domains | 74/85 (435/505) |
| 142 | 559 | 104642-106321 | Hypothetical protein *Bacillus* phage phiNIT1 YP_008318366.1 (566) /  DNA binding protein *Bacillus* phage Grass YP_008771467.1 (566) / CasRa_I-A domain | 57/75 (321/429)  57/76 (321/430) |
| 143 | 489 | 106337-107806 | Putative DNA helicase *Bacillus* phage SIOphi AGK86997.1 (488) / DnaB_C domain | 70/84 (336/407) |
| 144 | 347 | 107953-108996 | Nuclease SbcCD subunit D *Bacillus* phage SIOphi AGK86996.1 (362) / MPP_Mre11_N domain | 70/84 (248/299) |
| 145 | 632 | 109172-111070 | Putative exonuclease subunit 2 *Bacillus* phage SIOphi AGK86995.1 (631) / ABC_ATPase domain | 58/74 (365/471) |
| 146 | 198 | 111067-111663 | Hypothetical protein *Bacillus* phage vB_BceM-Bc431v3 YP_007677093.1 (190) | 44/60 (78/108) |
| 147 | 355 | 111666-112733 | DNA primase *Bacillus* phage SIOphi AGK86993.1 (356) / DnaG and ZnF_CHCC domains | 60/74 (214/265) |
| 148 | 212 | 112796-113434 | Putative deoxyuridine 5'-triphosphate nucleotidohydrolase *Bacillus* phage BCP78 YP_006908013.1 (210) / dUTPase domain | 62/78 (129/163) |
| 149 | 114 | 113452-113799 | Hypothetical protein SIOphi_00915 *Bacillus* phage SIOphi AGK86991.1 (118) | 31/46 (36/54) |
| 150 | 132 | 113803-114201 | Hypothetical protein *Bacillus* phage phiNIT1 YP_008318374.1 (128) | 47/69 (60/88) |
| 151 | 97 | 114188-114481 | Hypothetical protein GZ77_08910 *Endozoicomonas montiporae* KEQ14471.1 (108) | 35/49 (38/54) |
| 152 | 185 | 114481-115038 | Endonuclease *Bacillus* phage Grass YP_008771477.1 (184) /  Holliday junction resolvase *Bacillus* phage BigBertha YP_008771149.1 (183) / COG1591domain | 58/80 (107/149)  53/67 (97/123) |
| 153 | 790 | 115175-117547 | Ribonucleoside-diphosphate reductase, alpha subunit *Bacillus* phage vB_BceM-Bc431v3 YP_007677084.1 (779) / RNR_I domain | 68/80 (532/637) |
| 154 | 157 | 117639-118112 | HNH homing endonuclease *Enterococcus* phage IME_EF3 YP_009008925.1 (162) / NUMOD4 and HNH_3 domains | 41/57 (67/94) |
| 155 | 376 | 118204-119334 | Ribonucleotide reductase beta subunit *Bacillus* phage vB_BceM-Bc431v3 YP_007677082.1 (376) / RNRR2 and NrdF domains | 78/89 (292/337) |
| 156 | 71 | 119344-119559 | Hypothetical protein *Bacillus* phage phiNIT1 YP_008318385.1 (70) | 45/72 (31/50) |
| 157 | 263 | 119641-120432 | Hypothetical protein, partial *Bacillus* phage SPG24 BAO79562.1 (260) | 67/84 (172/216) |
| 158 | 102 | 120435-120743 | Hypothetical protein SIOphi_00855 *Bacillus* phage SIOphi AGK86979.1 (101) /  Putative integration host factor *Bacillus* phage phiNIT1 YP_008318387.1 (96) / HU_IHF domain | 76/89 (73/86)  65/81 (57/72) |
| 159 | 98 | 120830-121126 | Hypothetical protein *Bacillus* sp. UNC437CL72CviS29 WP_026593042.1 (81) | 37/52 (34/48) |
| 160 | 1057 | 121153-124326 | Putative DNA polymerase 2 *Bacillus* phage BCP78 YP_006907997.1 (1112)/ DNA_pol_A_pol_I_C, DNA_pol_A_exo1 and UDG_F4_TTUDGA_like domains | 59/72 (650/811) |
| 161 | 161 | 124423-124908 | Hypothetical protein *Bacillus* phage vB_BceM-Bc431v3 YP_007677068.1 (178) | 53/75 (83/118) |
| 162 | 400 | 125188-126390 | Hypothetical protein SIOphi_00810 *Bacillus* phage SIOphi AGK86970.1 (439) /  ssDNA binding domain protein *Bacillus* phage Bcp1 YP_009031420.1 (430) | 46/58 (203/256)  51/64 (172/216) |
| 163 | 89 | 126699-126968 | Hypothetical protein *Bacillus amyloliquefaciens* WP_025851624.1 (65) | 42/67 (25/40) |
| 164 | 381 | 127029-128174 | Recombinase A *Bacillus* phage SIOphi AGK86969.1 (403) / recA domain | 70/83 (258/304) |
| 165 | 117 | 128222-128575 | Hypothetical protein SIOphi_00800 *Bacillus* phage SIOphi AGK86968.1 (118) | 75/85 (85/97) |
| 166 | 211 | 128565-129200 | RNA polymerase sigma factor *Bacillus* phage Grass YP_008771504.1 (211) | 68/83 (143/176) |
| 167 | 97 | 129255-129548 | Putative holin protein *Bacillus* phage BCP78 YP_006907987.1 (109) / holin_SPP1 domain | 53/80 (43/65) |
| 168 | 157 | 129615-130088 | Hypothetical protein Grass_140 *Bacillus* phage Grass YP_008771506.1 (162) / PHA02283 domain | 48/67 (73/102) |
| 169 | 176 | 130099-130629 | Hypothetical protein *Flavobacterium johnsoniae* WP_012025189.1 (981) / Big_2 domain / Contains an amino acid repeat | N/A |
| 170 | 317 | 130767-131720 | Hypothetical protein SIOphi_00780 *Bacillus* phage SIOphi AGK86964.1 (315) | 51/71 (158/221) |
| 171 | 123 | 131771-132142 | Hypothetical protein Bcp1_148 *Bacillus* phage Bcp1 YP_009031429.1 (84) | 32/52 (27/45) |
| 172 | 435 | 132160-133467 | Metallophosphatase *Bacillus* phage Grass YP_008771509.1 (436) /  Putative DNA repair exonuclease *Bacillus* phage phiNIT1 YP_008318401.1 (436) / MPP_Mre11_N domain | 64/78 (276/341)  64/78 (276/340) |
| 173 | 129 | 133464-133853 | Hypothetical protein *Bacillus* phage phiNIT1 YP_008318402.1 (125) | 42/59 (50/71) |
| 174 | 204 | 133974-134588 | Hypothetical protein BCP78_0134 *Bacillus* phage BCP78 YP_006907969.1 (203) | 54/67 (103/128) |
| 175 | 241 | 134593-135318 | Hypothetical protein Grass_147 *Bacillus* phage Grass YP_008771513.1 (239) | 76/88 (179/209) |
| 176 | 182 | 135406-135954 | Hypothetical protein Grass_148 *Bacillus* phage Grass YP_008771514.1 (182) | 55/74 (99/133) |
| 177 | 174 | 136051-136575 | Hypothetical protein SIOphi_00745 *Bacillus* phage SIOphi AGK86957.1 (176) | 68/83 (117/145) |
| 178 | 286 | 136731-137591 | Hypothetical protein SIOphi_00740 *Bacillus* phage SIOphi AGK86956.1 (289) | 43/67 (122/194) |

^a^ Possible read through translation
