# Supplementary material for: Three novel bacteriophages isolated from the East African Rift Valley soda lakes
Source: Virol J. 2016 Dec 3;13:204. doi: 10.1186/s12985-016-0656-6 (PMC5135824; doi:10.1186/s12985-016-0656-6)
Supplement: Additional file 7: Figure S3. — GC skew analysis of the Mgbh1 genome showing putative replication origin (ori) and termination sites (ter) calculated using a window size of 1000 bp and a step size of 100 bp. (DOCX 22 kb) [file 12985_2016_656_MOESM7_ESM.docx]

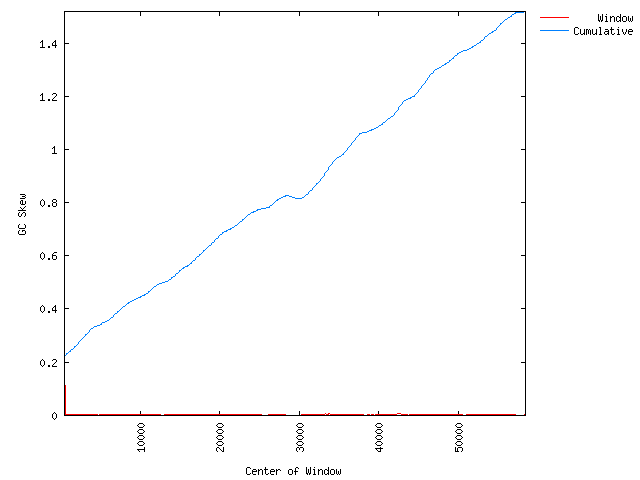
 **Figure S3.** GC skew analysis of the Mgbh1 genome showing putative replication origin (*ori*) and termination sites (*ter*) calculated using a window size of 1000 bp and a step size of 100bp
